# Tunable Terpolymer Series for the Systematic Investigation of Membrane Proteins

**DOI:** 10.1021/acs.biomac.4c01219

**Published:** 2024-12-26

**Authors:** Gestél C. Kuyler, Elaine Barnard, Pooja Sridhar, Rebecca J. Murray, Naomi L. Pollock, Mark Wheatley, Timothy R. Dafforn, Bert Klumperman

**Affiliations:** a Department of Chemistry and Polymer Science, Stellenbosch University, Private Bag X1, Matieland 7602, South Africa; b Centre for Health and Life Sciences, Coventry University, Coventry CV1 2DS, United Kingdom; c School of Biosciences, University of Birmingham, Edgbaston, Birmingham B15 2TT, United Kingdom; d Centre of Membrane Proteins and Receptors (COMPARE), University of Birmingham and University of Nottingham, Midlands B15 2TT, United Kingdom

## Abstract

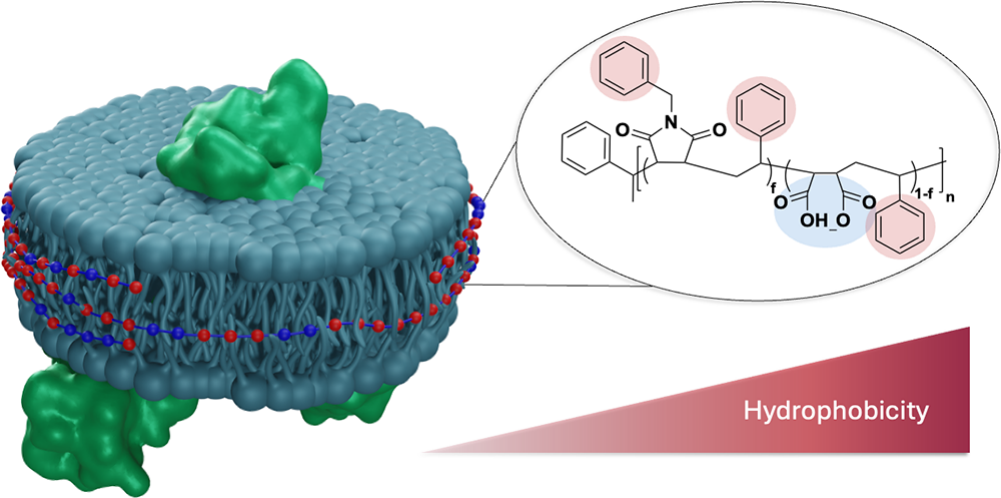

Membrane proteins (MPs) are critical to cellular processes and serve as essential therapeutic targets. However, their isolation and characterization are often impeded by traditional detergent-based methods, which can compromise their native states, and retention of their native lipid environment. Amphiphilic polymers have emerged as effective alternatives, enabling the formation of nanoscale discs that preserve MPs’ structural and functional integrity. We introduce a novel series of poly(styrene-*co*-maleic acid-*co*-(*N*-benzyl)maleimide) (BzAM) terpolymers with tunable amphiphilicity, synthesized through controlled polymerization. Designed to mimic and improve upon industry-standard poly(styrene-*co*-maleic acid), these well-defined terpolymers offer enhanced control over molecular weight and distribution, allowing for systematic evaluation of polymer properties and their effect on membrane solubilization. The BzAM series effectively solubilized membranes and demonstrated a direct correlation between polymer hydrophobicity and solubilization efficiency of bacterial ABC transporter, Sav1866. This research highlights the importance of rational polymer design in MP research and provides a foundation for future developments.

## Introduction

Membrane proteins (MPs) are essential diagnostic and therapeutic targets as they facilitate extra- and intracellular operations and account for various physiological functions in humans.^1^ A comprehensive understanding of an MP usually requires the MP to be solubilized and purified. However, the extraction of MPs from the cell membrane has posed several challenges compared to soluble proteins. Current methods predominantly rely on detergent-based approaches, which often compromise the functional and structural stability of the target protein, thereby impeding progress in MP research.^2^ In addition, preserving the structural integrity of MPs is paramount for accurate therapeutic design. Traditional detergents disrupt the lipid bilayer, solubilizing MPs into micellular structures to enhance water solubility.^3^ This process, however, strips the MPs of their native lipid environment. The lipid matrix in which MPs are embedded is both compositionally and biophysically complex.^4^ This complex lipid environment affects the structures, dynamics, and functions of MPs. In the case of many MP drug targets, the protein–lipid interface may be essential for therapeutic access to the binding sites within the protein,^5^ thus demonstrating the necessity of a more compositionally representative environment for full functionality.

Amphiphilic polymers have emerged as a valuable tool for MP research. In 2009, poly(styrene-*co*-maleic acid) (SMA) was shown to interact spontaneously with and penetrate the lipid bilayer, resulting in the formation of uniformly sized discoidal particles known as SMA lipid particles (SMALPs).^6^ A key advantage of this approach over other mimetic systems, such as amphipols^7^ and membrane-scaffold protein (MSP)^8^ nanodiscs, is the complete absence of detergents throughout the process. Polymer-mediated solubilization stabilizes MPs and their annular phospholipids within nanoscale discs, with the polymer located peripherally.^9^ This enables the analysis of MPs in a native-like environment while retaining their stability and physiological properties.^6^ Furthermore, polymer-stabilized nanodiscs offer compatibility with a wide range of microscopic, spectroscopic, and biophysical techniques.^10−17^

In recent years, SMA with a 2:1 styrene (hydrophobic) to maleic acid (hydrophilic) ratio has been deemed the “industry standard” for MP research.^18−20^ Despite its widespread adoption, this industrially used polymer suffers from inherent drawbacks related to the synthetic strategy employed in its production. Conventional radical polymerization results in broad molecular weight distributions, yielding a highly heterogeneous mixture of polymer chain lengths within a single sample.^21^ This variability may present limitations in methodical evaluation and hinder a comprehensive molecular understanding of polymer–lipid interactions.^22,23^

Recent efforts have increasingly focused on investigating polymer architecture and its impact on, *e.g*., lipid particle size, protein stability, and the conformational states of the stabilized MPs.^24−27^ A dedicated research domain has emerged to characterize commercially available polymers and expand the scope, with alternative polymers offering characteristics such as higher ionic strength, broader pH tolerance, and reduced Coulombic repulsion between membrane lipids and the polymer.^28,29^

The choice of solubilizing polymers is important, with research indicating that a “one-size-fits-all” approach is not viable for comprehending the structural and functional aspects of diverse protein types.^30^ The development of new amphiphilic polymers that retain the membrane solubilization capabilities of SMA while exhibiting varied physicochemical properties reflects the growing importance of polymer-stabilized nanodiscs in MP research. Achieving the interaction between the polymer and the cell membrane necessitates consideration of polymer properties such as hydrophobic/hydrophilic balance, overall composition, monomer sequence, molecular weight, and net charge.

This work utilized a controlled polymerization technique, i.e., reversible addition–fragmentation chain transfer (RAFT)-mediated polymerization, allowing for the rational design of polymers intended for MP isolation. A series of novel terpolymers are synthesized from an alternating base copolymer of poly(styrene-*alt*-maleic anhydride) (SMAnh) with well-defined chemical characteristics and narrow molecular weight distributions. The hydrophobic/hydrophilic balance of the copolymer was altered through the partial modification of its anhydride functionalities, resulting in an incremental increase in overall hydrophobicity throughout the series. The tunable amphiphilicity provides a unique platform for the systematic evaluation of polymer properties and their utility for membrane solubilization.

## Materials and Methods

### Chemicals and Reagents

Maleic anhydride (MAnh) briquettes (99%, Sigma-Aldrich) and azobis(isobutyronitrile) (AIBN) were purified by recrystallization from toluene and methanol, respectively, and dried in vacuo at ca. 25 °C overnight. The styrene (St) (99%, Sigma-Aldrich) monomer inhibitor was removed by passing through a basic aluminum oxide column prior to use. Benzylamine (99%, Sigma-Aldrich), 2-butanone (≥99%, Sigma-Aldrich), 1,3,5-trioxane (≥99%, Sigma-Aldrich), and *N,N*-dimethylformamide (DMF) (99.8%, anhydrous, Sigma-Aldrich) were used as received. For the RAFT agent *S*-butyl-*S*′-(1-phenyl ethyl)trithiocarbonate (BPT), 1-butanethiol (97%, Fluka), carbon disulfide (99%, Sigma-Aldrich), 1-bromoethylbenzene (97%, Sigma-Aldrich), and magnesium sulfate (anhydrous, ≥97%, Sigma-Aldrich) were used as received. Chloroform, pentane, and triethylamine (99%, Sigma-Aldrich) were distilled prior to use.

Milli-Q Millipore deionized water (pH 7.0) was used for all aqueous requirements. Deuterated solvents for analytical characterization included dimethyl sulfoxide ((CD_3_)_2_SO, 99.8%, MagniSolv) and acetone ((CD_3_)_2_CO, 99.9%, MagniSolv), which were used as received.

All other chemicals and reagents were purchased from Sigma-Aldrich unless stated otherwise. Sigma-Aldrich is a subsidiary of Merck KGaA.

The phospholipid 1,2-dimyristoyl-*sn*-glycero-3-phosphocholine (DMPC) was purchased from Avanti Polar Lipids (Alabaster, USA). Polymer controls include SMA2000, referred to as SMA2:1 (*M*_w_ 7500 g·mol^–1^, *Đ* 2.50, Cray Valley, USA), and RAFT-synthesized SMA1:1 (*M*_w_ 6800 g·mol^–1^, *Đ* 1.36).

### RAFT Agent Synthesis: *S*-Butyl-*S*′-(1-phenylethyl)trithiocarbonate (BPT)

The synthetic procedure for the BPT RAFT agent was adapted from the literature.^31^ In a 250 mL round-bottom flask, a solution of 1-butanethiol (5.05 g, 56.0 mmol) and carbon disulfide (8.53 g, 112 mmol) in 40 mL of chloroform was prepared and stirred at ca. 25 °C. During the dropwise addition of triethylamine (11.3 g, 112 mmol), the mixture changed from colorless to orange and was stirred for an additional 3 h at ca. 25 °C. 1-Bromoethylbenzene (10.4 g, 56.0 mmol) was added dropwise to the mixture, after which the reaction was stirred overnight under ambient conditions. The extent of the reaction was monitored by thin-layer chromatography (TLC). The reaction mixture was sequentially washed with deionized water (2 × 50 mL), 2 M H_2_SO_4_ (aq) (2 × 50 mL), deionized water (2 × 50 mL), and saturated brine solution (2 × 50 mL). The resulting solution was dried overnight by stirring over anhydrous MgSO_4_, followed by vacuum filtration and subsequent rotary evaporation to remove the residual solvent, yielding a dark-yellow/orange oil (14.8 g, 97.3% yield). Additional column chromatography on silica with 100% pentane eluent enhanced the purity.

Compound purity was determined to be 93% by ^1^H NMR spectroscopy (CDCl_3_). Peak assignments: δ 0.92 (t, 3H, C**H**_3_CH_2_CH_2_CH_2_–S), 1.42 (m, 2H, CH_3_C**H**_2_CH_2_CH_2_–S), 1.66 (m, 2H, CH_3_CH_2_C**H**_2_CH_2_–S), 1.76 (d, 3H, C**H**_3_(CH−)–S), 3.38 (t, 2H, – S–C**H**_2_), 5.37 (q, 1H, C**H**), 7.33 (m, 5H, Ar**H**).

### Base Copolymer: Poly(styrene-*alt*-maleic anhydride) (SMAnh)

Typical RAFT-mediated polymerization procedure is as follows:

In a 500 mL three-necked round-bottom flask fitted with an ice water condenser and oil bubbler, a solution of MAnh (36.8 g, 375 mmol), St (39.1 g, 375 mmol), BPT (4.06 g, 15.0 mmol), AIBN (0.493 g, 3.00 mmol), and 1,3,5-trioxane (1.22 g, 13.5 mmol) in 270 mL of 2-butanone was prepared. Copolymerizations were performed at a solid content of 30% (w/v) with a RAFT:initiator ratio of 5:1 and 1.5% (w/w) 1,3,5-trioxane as internal reference standard. The solution was degassed by purging with argon gas (45 min) while stirring. The reaction occurred at 80 °C for 20 h with stirring, after which it was quenched by cooling and exposure to atmospheric oxygen. The copolymer was isolated from the reaction mixture by precipitation in cold pentane (3 × 750 mL), followed by vacuum filtration. The resultant copolymer was dried *in vacuo* at 40 °C overnight to yield a fine pale-yellow powder (79.6 g, 99.0% yield).

### RAFT *S-*Butyl Trithiocarbonate Removal via Thermolytic Cleavage

The thermal stability of SMAnh was evaluated using thermogravimetric analysis (TGA) (experimental details to follow). Pale-yellow SMAnh powder was placed in a 500 mL single-neck round-bottom flask. The polymer was heated to 200 °C under vacuum while rotating in a silicon oil bath for 5–7 h. The thermolysis reaction was monitored by periodic UV analysis (1 mg·mL^–1^, DMF solvent), and the reaction time was adjusted according to the amount of material subjected to thermolysis. Generally, complete end-group removal of 50 g of SMAnh was achieved after 5 h at 200 °C under vacuum. After the polymer cooled to ca. 25 °C, it was purified by dissolution in acetone (30% (w/v)) and precipitation in excess cold pentane, followed by drying *in vacuo* overnight at 40 °C to yield an off-white powder.

### General Synthesis of Poly(styrene-*co*-maleic anhydride-*co*-(*N*-benzyl)maleimide) (BzAM)

Typically, 12.0 g (61.2 mmol MAnh) of SMAnh (end-group removed) was dissolved in 48 mL of DMF (25% (w/v)) in a three-necked 250 mL round-bottom flask fitted with an oil bubbler under magnetic stirring. To produce the BzAM series, varying amounts of benzylamine (Table S1) were mixed with a small amount of DMF before dropwise addition to the solution. The 0.05 BzAM derivative exemplifies a typical reaction: benzylamine (0.333 g, 3.11 mmol) was mixed in 2 mL of DMF and added dropwise to the SMAnh solution. The reaction mixture was stirred at 30 °C for 3 h to produce the ring-opened version of the terpolymer. The reaction mixture was subsequently heated to 130 °C and stirred for 5 h. This heating step resulted in ring-closed maleimide functionalities. The terpolymers were isolated from the reaction mixture by removal of DMF under rotary evaporation. The resultant polymers were redissolved in 100 mL of acetone, followed by rotary evaporation until nearly dry. This step facilitated the effective removal of excess DMF. The polymers were further purified through acetone dissolution (∼20% (w/v)) and precipitation in cold diethyl ether (3 × 300 mL), followed by centrifugation (10 min, 3300 × *g*). The resulting terpolymers were dried overnight *in vacuo* at 40 °C to yield an array of light-yellow powders.

### General Hydrolysis Procedure Poly(styrene-*co*-maleic acid-*co*-(*N*-benzyl)maleimide) (BzAM)

Hydrolysis of the remaining MAnh repeat units was achieved under standard reflux conditions using a setup consisting of a 100 mL round-bottom flask, fitted with an ice water condenser and an oil bubbler. 2.00 g of the terpolymer was suspended at 10% (w/v) in a 1 M NaOH solution (20 mL, 20.0 mmol) and heated to 100 °C while stirring for 6 h. Initially, the terpolymer formed a suspension of solids in the aqueous solution. Upon completion of the hydrolysis reaction, a clear yellow/light-brown solution was obtained.

After cooling, the resultant maleic acid-containing product was purified by dialysis (3500 MWCO) against dH_2_O for 48 h with regular water changes. Drying by lyophilization yielded an array of powders with yellowish hues.

Alternatively, hydrolysis can be achieved using autoclaving. This technique allows for reduced reaction time, compared to traditional reflux methods.^32^ In a 250 mL Schott bottle, 2.00 g of terpolymer was suspended in a 10% (w/v) 1 M NaOH solution (20 mL, 20 mmol). The reaction mixture in the loosely capped bottle was subjected to two standard autoclave sterilization cycles (20 min at 121 °C). After cooling, the resultant product was purified by dialysis (3500 MWCO) against dH_2_O for 48 h with regular water changes. Drying by lyophilization yielded an array of powders with a yellowish hue.

### Preparation of Polymer Stock Solutions

Polymer stock solutions were prepared at 10% (w/v) (BzAM terpolymers, SMA1:1, and SMA2:1) in pure dH_2_O, kept at 4 °C, and protected from light. The polymer stocks were diluted in the respective buffers as indicated in the individual experiments.

#### SMAnh Copolymer and BzAM Terpolymer Characterization

##### Nuclear Magnetic Resonance (NMR) Spectroscopy

The ring-closed (anhydride) polymers were characterized by liquid-state ^1^H and ^13^C NMR spectroscopy using either deuterated acetone or DMSO on a Varian VXR-Unity/Agilent (300 MHz) or Bruker Ascend (400 MHz) at 298 K. Quantitative ^13^C NMR spectra were obtained by implementing specialized conditions (Table S2). MestReNova (12.0.3) computer software was used for data processing.

##### Attenuated Total Reflectance (ATR) Fourier Transform Infrared (FTIR) Spectroscopy

BzAM derivatives were characterized by ATR-FTIR using a Thermo Nicolet iS10 Smart iTR spectrometer with a diamond/ZnSe internal reflection crystal ATR accessory. Spectra were recorded from 600 to 4000 cm^–1^ with a spectral resolution of 4 cm^–1^, utilizing 64 individual scans. Transmittance (%) was normalized between 0 and 100%.

##### Size Exclusion Chromatography (SEC)

DMF-SEC analysis utilized a mobile phase of *N*,*N*-dimethylformamide (DMF) (Sigma-Aldrich, Chromasolv Plus, for HPLC ≥99.9%) stabilized with 0.05 M LiBr. The SEC setup consisted of a Waters 717plus autosampler with a Waters in-line degasser AF connected to a Shimadzu LC-10AT pump. The column setup consisted of a precolumn (1 × PSS GRAM column) with a 10 μm particle size and dimensions of 8.0 × 50 mm, analytical columns 1 × PSS GRAM (10 μm particle size, 100 Å pore size, and 8.0 × 300 mm), and 2 × PSS GRAM columns (10 μm particle size, 3000 Å pore size, and 8.0 × 300 mm). A Waters 410 differential refractometer and Waters 2487 dual-wavelength absorbance detector were connected in series. The flow rate was 0.8 mL/min, and the columns were kept at 40 °C. Samples were prepared at 2 mg·mL^–1^ in DMF (0.05 M LiBr) and filtered (0.45 μm pore size, PTFE) before analysis. The SEC system was calibrated using low-dispersity poly(methyl methacrylate) (PMMA) calibration standards. All molecular weight and dispersity values are reported as PMMA equivalents. Agilent GPC/SEC software was used to determine the experimental molar mass (*M*_n__,__SEC_) and dispersity (*Đ*) values through conventional PMMA calibration.

##### Thermogravimetric Analysis (TGA)

TGA was conducted using a TA Instruments Q500 system. Samples were housed in aluminum pans and purged at a flow rate of 50.0 mL·min^–1^ under nitrogen (N_2_) gas. Thermal degradation studies were conducted from 25–600 °C at a constant heating rate of 10 °C·min^–1^.

##### Ultraviolet/Visible Light (UV/Vis) Spectroscopy

UV/vis spectra were obtained using an Analytik Jena SPECORD 210 PLUS UV/vis spectrophotometer in the wavelength range of 190–1100 nm, with a minimum of three measurements per sample. Samples were prepared at a concentration of 1 mg·mL^–1^ in DMF to monitor the presence of the RAFT end group at 310 nm.

##### Preparation of Large Unilamellar Vesicles (LUVs)

Lyophilized DMPC was suspended in dH_2_O at a concentration of 10 mg·mL^–1^. The lipids were resuspended using vortex mixing and sonication in a water bath (10 min) to achieve a thoroughly mixed dispersion. The LUV (or liposome) dispersion was separated into 1 mL aliquots and extruded by 11 passes of the solution through 200 nm nucleopore track-etched polycarbonate membranes (Avanti Polar Lipids, USA). Dynamic light scattering (DLS) confirmed the 200 nm size of the DMPC LUV. A final LUV concentration of 1.25 mg·mL^–1^ was used in the subsequent experiments.

##### Preparation of Lipid-Only Polymer Nanodiscs

Polymer solutions (10% (w/v)) were added to an equal volume of DMPC LUV to obtain a final concentration of 1.25 mg·mL^–1^ DMPC and 2.5% (w/v) polymer. Polymer–DMPC LUV mixtures were incubated (16 h, ca. 25 °C) prior to analysis.

##### DLS

DLS was used to measure the *Z*-average diameter (nm) and size distribution profiles of lipid-only polymer nanodiscs. DLS experiments were performed using a DynaPro Plate Reader III and Dynamics software (Wyatt Technology, Haverhill, UK), using the laser wavelength of 825.4 nm with a detector angle of 150°. Each sample (60 μL) was loaded into a 96-well plate glass-bottom SensoPlate (Greiner Bio-One, Germany) in triplicate. Each measurement consisted of 10 scans of 10 s, carried out at 25 °C, with the attenuator position and laser power automatically optimized for size determination (nm).

DLS measurements of polymer-only (pH stability) samples were performed on a Zetasizer Nano-S ZEN1600 (Malvern Instruments, Malvern Panalytical, UK) and Zetasizer software, working with a 633 nm He–Ne laser and a detection angle of 90°. Samples (1 mL) were equilibrated at 25 °C for 2 min before measurements were performed in a quartz glass cuvette. Four repeat measurements were taken of each sample and consisted of 20 scans of 10 s, carried out at 25 °C, with the attenuator position and laser power automatically optimized for size determination (nm).

##### Potentiometric Titrations

Potentiometric titrations enabled the determination of the effective p*K*_a_ values of the various polymers. Polymers were prepared in dH_2_O at 0.3% (w/v), adjusted to pH 12 (1.0 M NaOH). The temperature was kept at 25.8 ± 0.2 °C using a temperature-controlled circulatory water bath. The backward titration of the anionic BzAM series was carried out with standardized 0.1 M HCl. All titration experiments were repeated at least twice. 907 Titrando Autotitrator (Metrohm, SA) with 50 mL Dosino dosing units, mechanical overhead stirring, Syntrode (combined pH electrode and integrated Pt1000 temperature sensor), and Tiamo V2.4 software were used to conduct and monitor the titrations. The Syntrode was referenced against 0.1 M KCl and calibrated using pH calibration buffers at pH 4.0, 7.0, and 12.0 (Metrohm, SA). Equivalence points (EQP) were calculated according to the first derivative of the titration curve, i.e., the Equivalence Point Recognition Criterion (ERC) function determined by Tiamo V2.4 software. For polyprotic acids, additional p*K*_a_ may be observed halfway between two consecutive EQPs, i.e., the half equivalence point (1/2 EQP). In the case of SMA and BzAM terpolymers, three EQPs and two 1/2 EQPs were identified, where the first EQP corresponds to the titration of excess NaOH and the two 1/2 EQPs correspond to the two disparate p*K*_a_ values of the maleic acid repeat units.

##### Divalent Cation and pH Stability

The divalent cation stability of the lipid-only polymer nanodiscs under varying CaCl_2_ or MgCl_2_ concentrations (0–25 mM) in buffer (50 mM Tris, pH 8.0) was evaluated using optical density measurements at 630 nm (UV/vis spectrophotometer, Anthos Zenyth 200rt Microplate Reader, Biochrom). Final concentrations of 0.5% (w/v) polymer and 1.25 mg·mL^–1^ DMPC were used. The tolerable concentration range was determined as the minimum concentration of MgCl_2_ or CaCl_2_ added before the OD measurements exceeded 0.1 A.U.

The pH stability of the lipid-only polymer nanodiscs was evaluated using various pH buffers and literature-reported methods,^3^ analyzed by DLS (DynaPro Plate Reader III).

The pH stability of polymer-only solutions was evaluated by automated titration using a 907 Titrando Autotitrator. The pH was adjusted with NaOH and HCl, and 1 mL aliquots were collected at specific pH points. All samples were maintained at a final polymer concentration of 0.5% (w/v) in 150 mM NaCl, analyzed by DLS (Zetasizer Nano-S ZEN1600). The stable pH range (Table 3) was determined as the minimum pH where the standard deviation (SD) of the hydrodynamic diameter (nm) as a proportion of volume exceeded 35% error. The marked increase in diameter and error is indicative of polymer aggregation and/or precipitation. These values were corroborated with the determined effective p*K*_a_.

##### Transmission Electron Microscopy (TEM)

Nanodisc-containing fractions were selected for negative-stain TEM following SEC using a Superdex 200 10/300 GL gel filtration column at a flow rate of 0.5 mL·min for 1.2 column volumes, pre-equilibrated in 50 mM Tris, 150 mM NaCl at pH 7.4. DMPC nanodiscs (5 μL) adsorbed to glow discharged Formvar carbon-coated copper grids (300 mesh) by 2 min incubation. The excess sample was removed using filter paper, followed by a 10 s wash step with dH_2_O, where excess water was removed with filter paper. Samples were negatively stained using 5 μL of 2% (w/v) uranyl acetate, followed by a 1 min incubation and excess stain removal. Grids were left to air-dry prior to imaging with a Jeol 2100 Plus TEM at 200 kV. Samples were imaged at ×80,000 magnification, indicating 100 nm scale bars.

##### Preparation of Protein-Containing *Escherichia coli* Membranes

Putative multidrug export ATP-binding/permease protein (Sav1866) was used for the solubilization experiments. The plasmid (pET) expressing the protein of interest was transformed into BL21(DE3) (New England Biolabs) using a standard transformation protocol.^33^ Cultures were grown in Luria Broth (LB) (10 g of tryptone, 5 g of yeast extract, 10 g of NaCl in 1 L of dH_2_O) supplemented with 100 μg·mL^–1^ of ampicillin (Melford Laboratories Ltd., UK) until the optical density (OD 600 nm) of the cell suspensions reached 0.4–0.6 A.U. Protein expression was induced by the addition of 0.5 mM isopropyl β-d-1-thiogalactopyranoside (Melford Laboratories) for 16 h at 18 °C.

The cultures were subsequently harvested at 5000 × *g* (15 min, 4 °C) (Beckman coulter JXN centrifuge, JLA8.1000 rotor), resuspended in 50 mM Tris, 150 mM NaCl, pH 8.0, and then mechanically lysed using the Avestin C3 Cell Disrupter by three passages at 17,000 PSI. The lysate was centrifuged (Beckman Coulter JXN centrifuge, JA25.50 rotor) at 10,000 × *g* for (30 min, 4 °C) to isolate the cell debris. The resulting supernatant was further subjected to ultracentrifugation at 100,000 × *g* (1 h, 4 °C) (Beckman Coulter 70 Ti fixed angle rotor) to isolate the protein-containing membrane fractions. The membrane fractions were resuspended in purification buffer (50 mM Tris, 150 mM NaCl, pH 8.0) at a wet-pellet weight of 80 mg·mL^–1^ and stored at −80 °C until further use.

##### Solubilization of MPs from *E. coli* Membranes

Polymer solutions of 5.0% (w/v) were added to equal volumes of Sav1866 membranes (80 mg·mL^–1^), yielding final membrane concentrations of 40 mg·mL^–1^ and 2.5% (w/v) polymer. Membrane-polymer solutions were incubated at ca. 25 °C for 2 h.

Following polymer-mediated solubilization, the samples were subjected to ultracentrifugation at 100,000 × *g* for 30 min (Beckman Coulter 70Ti fixed angle rotor) at 4 °C to separate the polymer-stabilized nanodiscs in the soluble fraction from the insoluble or sedimented fraction. The insoluble fractions were resuspended to the same volume in the purification buffer (50 mM Tris, 150 mM NaCl, pH 8.0). Crude membrane fractions were used as reference samples on the respective gels. The crude membrane fractions were diluted to comparable concentrations (containing no solubilization agent) and analyzed alongside the polymer solubilized sample fractions for relative densitometric comparison.

##### Affinity Purification of Sav1866 Polymer Nanodiscs

Polymer-mediated solubilization (40 mg·mL^–1^ membrane concentration, 2.5% (w/v) polymer) and subsequent ultracentrifugation (30 min, 100,000 × *g*) yielded the soluble fraction of the Sav1866 nanodiscs. For affinity purification, the soluble fraction was bound to 50 μL of equilibrated nickel(II)-nitrilotriacetic acid (Ni-NTA) agarose beads (Qiagen, Cat. No. 30210) overnight at 4 °C. The flow through was collected, and the beads were washed with purification buffer (50 mM Tris, 150 mM NaCl, pH 8.0) supplemented with 20 mM imidazole. The protein was eluted with the same buffer (50 mM Tris, 150 mM NaCl, pH 8.0) containing 500 mM imidazole. Immunoblotting and densitometric analysis of the soluble, insoluble, and eluted fractions were conducted as described below.

##### Polymer Dose–Response Experiments

Two BzAM derivatives (0.15 BzAM and 0.35 BzAM) were used for initial dose–response experiments at three different final polymer concentrations (0.5, 2.5, and 5.0% (w/v)). Briefly, the membrane fraction overexpressing Sav1866 was subjected to polymer-mediated solubilization using the different polymer concentrations, followed by a small-scale Ni-NTA purification as described above. Immunoblotting and densitometric analysis of the eluted fractions were conducted to determine the protein yield in each case.

#### Target Protein Identification by Gel Electrophoresis and Immunoblotting

##### Gel Electrophoresis

Samples for gel electrophoresis were prepared using the soluble, insoluble, or eluted fractions following polymer-mediated solubilization. Sample volumes of 60 μL were mixed with 20 μL of 4× LDS loading buffer (NuPAGE LDS sample buffer, Thermo Fisher Scientific). Polyacrylamide gel electrophoresis (PAGE) was carried out by loading sample volumes of 2 μL onto 4–15% Criterion TGX gels (Bio-Rad, 4561086 or 5671085), along with 5 μL of PeqGold VI protein marker (VWR). A diluted crude membrane fraction (no polymer addition) was used as a reference alongside the soluble, insoluble, and eluted fractions to allow for relative densitometric quantification. Tris-glycine buffer (Bio-Rad) was used as the running buffer during analysis. A voltage of 180 V was applied for 40 min, while monitoring the blue-stained buffer front. The gels were stained using Quick Coomassie (Generon) following stain incubation (2 h, *ca*. 25 °C) and dH_2_O destaining overnight.

##### Immunoblotting

Following PAGE, gels were transferred to poly(vinylidene fluoride) (PVDF) membranes using a Transblot-Turbo (Bio-Rad) for immunoblotting. The blots were blocked (16 h, 4 °C) with 5% (w/v) skimmed milk powder (VWR), followed by 1 h incubation with an anti-His primary antibody (Takara Bio, Japan) (1:5 000 in TBST (1× Tris-buffered saline (TBS) with 0.1% Tween20 (Sigma-Aldrich)) at ca. 25 °C. The blots were subsequently washed (3×) with TBST and incubated with goat antimouse IgG H&L (HRP) (ab205719, Abcam) (1:10 000 in TBST) for 1 h at ca. 25 °C. Finally, the blots were washed (3×) with TBST and incubated (60 s) with Amersham ECL Western Blotting Detection Agent (Cytiva) immediately prior to visualization on an Amersham Imager 600 (Cytiva). Sav1866 appeared at 65 kDa on PAGE, and the identity of this band was confirmed using target-specific antibodies against the *N-*terminal polyhistidine (His)-tagged protein.

##### Densitometric Analysis

*E. coli* membranes overexpressing Sav1866 were used to assess the membrane solubilization capabilities of the BzAM terpolymers and SMA controls. Following immunoblotting, the blots were scanned in grayscale. Relative densitometric integration of the sample bands allowed for the quantification of Sav1866 compared to the crude membrane fraction (M) using ImageJ 1.54g software.

## Results and Discussion

### Synthesis and Characterization of the Base Copolymer

**Scheme 1:**
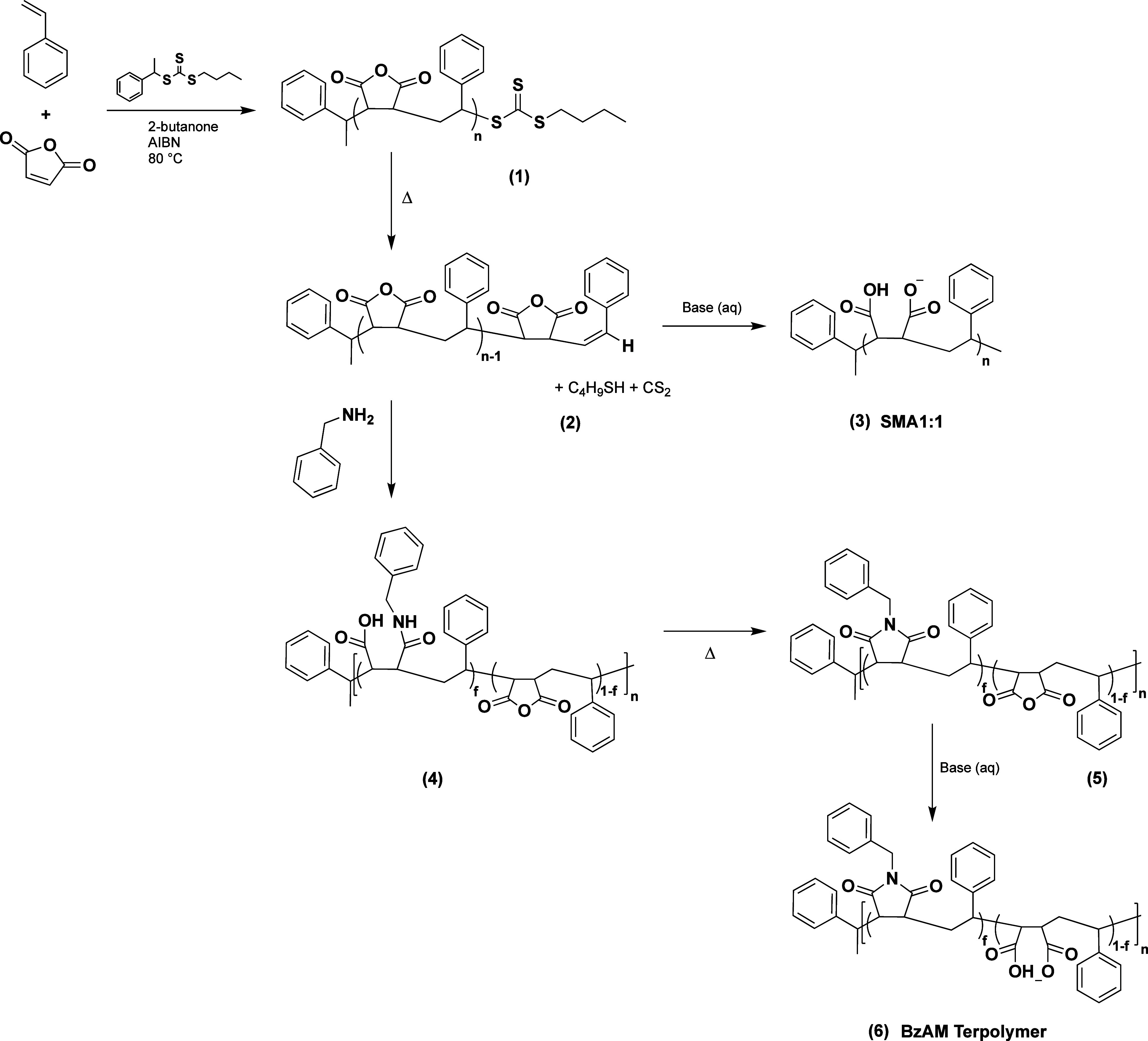
Synthetic Overview of RAFT-Mediated Copolymerization of Poly(styrene-alt-maleic anhydride) (SMAnh) (1), Subjected to Thermolytic Cleavage of the *S*-Butyl Trithiocarbonate End Group (2), Followed by Either Basic Hydrolysis to Yield Poly(styrene-alt-maleic acid) (SMA1:1), (3) or Post-Polymerization Modification with Varying Molar Amounts of Benzylamine, (4) and Thermal Dehydration to Yield Poly(styrene-co-maleic anhydride-co-(*N*-benzyl)maleimide) (5). Finally, Basic Hydrolysis Yields the Water-Soluble Poly(styrene-co-maleic acid-co-(*N*-benzyl)maleimide) (BzAM) Terpolymer (6) Series

A novel series of terpolymers emanating from an alternating base copolymer of poly(styrene-*alt*-maleic anhydride) (SMAnh), with well-defined chemical characteristics and narrow molecular weight distributions, was synthesized according to Scheme 1. This was achieved through a controlled radical polymerization technique, i.e., reversible addition–fragmentation chain transfer (RAFT)-mediated polymerization.

The RAFT *S-*butyl trithiocarbonate end group was thermolytically cleaved post-polymerization to yield unsaturated carbon–carbon double bonds at the ω-chain ends. These functional chain ends allow for uncomplicated backbone functionalization and enable further chain-end modification.

The water-soluble hydrolysis product of SMAnh, poly(styrene-*alt*-maleic acid) (SMA), has been reported to be too hydrophilic for effective membrane solubilization.^34^ This problem was overcome by altering the hydrophobic/hydrophilic balance of the alternating copolymer to produce a series of chemically modified terpolymers.

A range of sequential modifications of available maleic anhydride (MAnh) comonomer repeat units with varying molar amounts of benzylamine allowed for an incremental increase in the hydrophobic character throughout the terpolymer series.

^1^H NMR spectroscopy is widely employed for the characterization of polymers. However, in the case of SMAnh, a broad peak overlap is prevalent, and an alternative NMR technique is required. Specialized conditions were employed to obtain quantitative ^13^C NMR spectroscopic analyses (Table S2), notably increased relaxation delays, and the use of inverse-gated decoupling.^35^ The alternating microstructure of the base copolymer could be confirmed by ^13^C NMR spectroscopy. The relative chemical shift of the aromatic quaternary carbon of styrene was used to determine the local neighboring repeating unit, the styrene-centered triad of MSM, with the main peak appearing at 135.7–141.5 ppm. The lack of peaks in the regions 141.5–144.5 and 144.5–148.0 ppm reiterates the absence of random (SSM) and homopolystyrene (SSS) sequences, respectively, in SMAnh.^36^ Thus, confirming a well-defined, alternating base copolymer was synthesized (Figure S1). The number-average molecular weight (*M*_n_) and molecular weight dispersity (*Đ*) were determined by size exclusion chromatography (SEC) and are summarized in Table 1.

**Table 1:** Summary of Conversion (α) and Molecular Weight (*M*_n_) Data of the SMAnh Base Copolymer

sample	*M*_n,target_ (g·mol^–1^)	overall **α**^a^**(%)**	*M*_n,theo_^b^ (g·mol^–1^)	St **α**^a^**(%)**	MAnh **α**^a^**(%)**	*M*_n,SEC_^c^ (g·mol^–1^)	*Đ*^c^
SMAnh	5300	99	5300	100	99	5000	1.36

a Conversion (α) was determined by relative peak integration using ^1^H NMR spectroscopy.

b Theoretical *M*_n_ calculated using monomer conversions.

c Molecular weight and dispersity values were obtained by size exclusion chromatography (SEC) with DMF as mobile phase and poly(methyl methacrylate) (PMMA) calibration standards.

### RAFT *S-*Butyl Trithiocarbonate End-Group Removal

To enable the systematic investigation of the effect of incremental changes in the amphiphilicity of the polymer on its membrane solubilization ability, the *S*-butyl trithiocarbonate end group (*i.e*., the end group (EG)) was cleaved to limit the number of factors that may influence the solubilization efficiency. Moreover, end-group removal is required to limit side reactions and complications during subsequent modifications.

Thermolysis has several advantages, particularly the lack of chemical additives to cleave the trithiocarbonate. The effective employment of this technique does, however, rely on the thermal stability of the polymer and desired functionalities.^37^ Thermolysis has been shown to be a simple and effective end-group removal strategy resulting in unsaturated chain ends that may facilitate subsequent modifications or the production of macromonomers with narrow molecular weight distributions.^38^

The thermolytic cleavage of *S*-butyl trithiocarbonate and the thermal stability of the copolymer were examined by thermogravimetric analysis (TGA) (Figure S2). The initial mass loss step below 100 °C can be attributed to the loss of polymer-bound water or small amounts of residual solvent present in the sample prior to analysis.^39^ The first mass loss step of interest (Figure S2A) with an onset temperature of 215 °C corresponds to a total mass loss of 2.8%, which correlates well with the theoretically expected mass loss of 3.3% for complete removal of the *S*-butyl trithiocarbonate (C_5_H_10_S_3_, 166.33 g·mol^–1^), assuming that nearly all the polymer chains contain the trithiocarbonate moiety. The second major mass loss step (Figure S2B), starting at 270 °C, corresponds to the onset of polymer degradation, marked by the evolution of carbon dioxide (decarboxylation), with the degradation maximum observed at 380 °C. These observations concur with literature reports on the thermolytic cleavage of trithiocarbonates and the thermal degradation of alternating SMAnh.^40−43^

In all cases, the temperature corresponding to mass loss associated with the cleavage of the trithiocarbonate group was lower than and largely independent of the main polymer degradation temperature, as determined by TGA. The thermolysis product was subsequently characterized by SEC (Figure S3A), UV–vis (Figure S3B), and ^1^H NMR spectroscopy (Figure S4), confirming the integrity of the base copolymer following successful end-group cleavage with an insignificant effect on copolymer molecular weight distributions.

### Synthesis and Characterization of the Terpolymer Series

The reactivity of the MAnh repeat units was exploited to systematically alter the degree of modification and thus the overall amphiphilicity of the resultant terpolymers. Incremental modification of the SMAnh base copolymer with benzylamine produced poly(styrene-*co*-maleic anhydride-*co*-(*N*-benzyl)maleimide). The successful conversion of SMAnh to a ring-closed series of BzAM derivatives was confirmed by ^1^H NMR (Figure S5) and ATR-FTIR (Figure S6). All BzAM derivatives result from the partial conversion of the anhydride moieties of SMAnh and are characterized by the reduced peak intensities of the carbonyl stretching vibrations at 1851 cm^–1^ (symmetric stretching) and 1767 cm^–1^ (asymmetric stretching) and the reduced peak intensity of the cyclic anhydride C–O–C stretching bands between 1285 and 860 cm^–1^. Subsequent maleimide formation is confirmed by the partial shift of the C=O carbonyl peak to approximately 1650 cm^–1^. An additional sharp peak at 1387 cm^–1^ appears for all the BzAM derivatives corresponding to the C–N stretch of the newly formed imide moiety.

Quantitative ^13^C NMR spectroscopy (Figure S7) was used to quantify the degree of benzylamine modification through relative peak integration. The percentage modification (Table 2) aligns closely with the targeted modifications, resulting in a series of terpolymers characterized by increasing molar amounts of hydrophobic moieties (*f*_BzAm_ and *f*_St_) and a decrease in the hydrophilic fraction (*f*_MAnh_).

**Table 2:** Effective p*K*_a_ Values of the BzAM Terpolymer Series as a Function of Benzylamine Functionalization, where the Calculated Monomer Molar Fractions (*f*_*x*_) Are Derived from the Percentage of Benzylamine (mol.% BzAM) Functionality Determined by Quantitative ^13^C NMR Spectroscopy^a^

sample	p*K*_a1_	p*K*_a2_	mol.% BzAM^b^	*f*_BzAM_	*f*^c^_hydrophobic_	*f*^d^_hydrophilic_
SMA2:1	5.29 (±0.01)	8.90 (±0.03)	0	0	0.645^e^	0.355^e^
SMA1:1	4.38 (±0.03)	9.02 (±0.02)	0	0	0.500	0.500
0.05 BzAM	5.33 (±0.02)	9.02 (±0.06)	2.9	0.015	0.515	0.485
0.10 BzAM	5.40 (±0.03)	8.95 (±0.01)	10.0	0.050	0.550	0.450
0.15 BzAM	5.67 (±0.03)	9.09 (±0.03)	13.9	0.070	0.570	0.430
0.20 BzAM	5.90 (±0.01)	9.09 (±0.07)	23.3	0.117	0.617	0.383
0.25 BzAM	5.68 (±0.01)	9.17 (±0.02)	27.6	0.138	0.638	0.362
0.30 BzAM	5.81 (±0.03)	8.95 (±0.03)	31.3	0.157	0.657	0.343
0.35 BzAM	6.09 (±0.04)	9.08 (±0.02)	36.6	0.183	0.683	0.317
0.40 BzAM	6.23 (±0.03)	9.21 (±0.03)	39.5	0.198	0.698	0.302

a Data are shown as mean ± SD (*n* = 2) (25.8 °C ± 0.2 °C).

b Percentage (%) modification determined through quantitative ^13^C NMR relative peak integration.

c Total hydrophobic molar fraction (_hydrophobic_) consisting of f_BzAM_ (*N*-benzyl maleimide fraction) and/or *f*_St_ (styrene fraction).

d Total hydrophilic fraction consisting of maleic acid fraction (*f*_MAc_).

e Molar fractions derived from the acid number reported by the manufacturer.^46^

Basic hydrolysis (aq. NaOH) yielded the anionic water-soluble series of aromatically substituted SMA-like terpolymers (see Figure S8 for ATR-FTIR spectra). Dissolution of the 0.45 and 0.50 BzAM derivatives was not achieved during the hydrolysis process, which indicated the upper hydrophobic limit of this series.

### Polymer Solution Behavior and Synthetic Membrane Solubilization

#### Effective p*K*_a_ and Polymer pH Dependence

**Figure 1:**
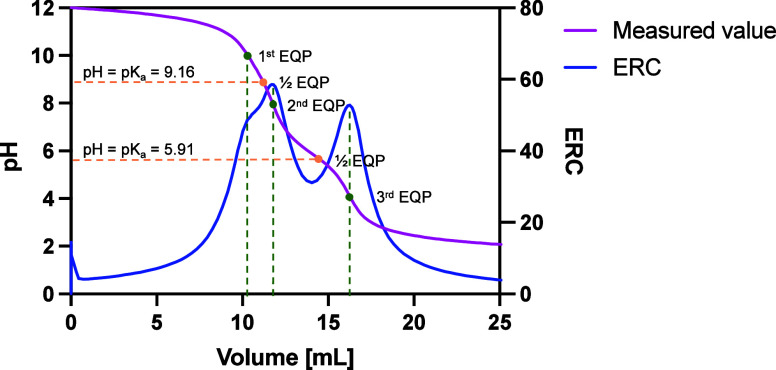
Exemplary potentiometric titration curve (magenta) and ERC (blue) of 0.20 BzAM (0.3% (w/v)) with a strong acid (1.0 M HCl). The respective equivalence points (EQP) are indicated on the curves and highlight the p*K*_a_ approximations.

Understanding the aqueous solubility of amphiphilic polymers is crucial for assessing their applicability in membrane solubilization under specific experimental conditions. One key factor to consider is the solution pH, which determines the charge density of the polymers. The pH range in which SMA and BzAM remains soluble is expected to vary depending on the polymer composition, as alterations in the styrene/*N*-benzylmaleimide-to-maleic acid ratio affect both the overall hydrophobicity and maximum number of charges on the polymer.^25^ The extent of ionization depends on the acid strength (p*K*_a_) of its acid moieties.

p*K*_a1_ is associated with the deprotonation of the first carboxyl group and, thus, the stronger of the two acids. A distinct effect on acid strength (p*K*_a1_) is observed as a function of functionalization, as the p*K*_a_ values are influenced by neighboring hydrophobic units and negative charges.^44^ Therefore, the different anionic BzAM derivatives can exhibit distinct p*K*_a_ values and, consequently, variation in ionization states at a given pH. Figure 1 shows representative potentiometric titration curves of the BzAM series (full series in Figure S9). Table 2 summarizes the effective p*K*_a_ values determined by potentiometric titrations as a function of benzylamine modification. As expected, SMA and the BzAM derivatives exhibit two effective p*K*_a_ values due to the presence of the diacid functionality. Alternating SMA1:1 with the greatest overall number of diacid moieties exhibits the lowest p*K*_a1_ of 4.38 (±0.03), while SMA2:1 exhibits an increased p*K*_a1_ of 5.29 (±0.01). As the degree of benzylamine functionalization in the terpolymer series increases, the p*K*_a1_ values gradually increase throughout the series, signifying reduced acid strength. This occurrence may be attributed to the influence of the overall increase in hydrophobic units, the distribution of acidic units, and the proximity of charges along the polymer backbone.^45^ Throughout the BzAM series, a slight but gradual increase in the value of p*K*_a1_ is observed, indicating a highly functional terpolymer series that allows fine-tuning of polymer amphiphilicity.

The molecular conformation and solubility of SMA and BzAM are greatly affected by the pH-dependent protonation/deprotonation state of the maleic acid repeat units. At neutral or high pH (above the p*K*_a_), electrostatic repulsion between the two carboxylate moieties dominates the hydrophobic effect. This leads to a random coil conformation and dissolution in aqueous media. Decreasing the solution pH below the p*K*_a_ of maleic acid results in complete protonation of the charged moieties. The loss of electrostatic repulsion promotes conformational change to a globular conformation driven by hydrophobic effects, resulting in polymer aggregation and eventual precipitation.^25,47^

**Figure 2:**
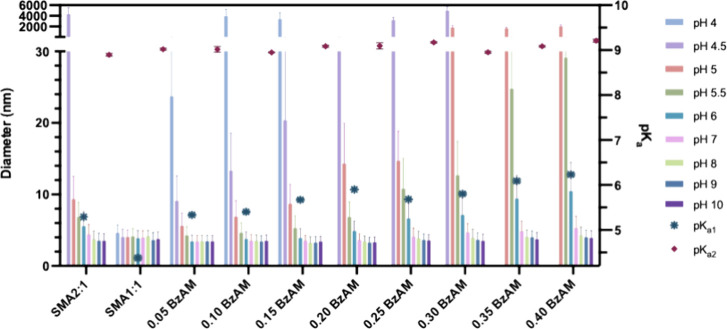
Average hydrodynamic diameter of polymer-only solutions determined as a proportion of volume by DLS as a function of pH. Final polymer concentration of 0.5% (w/v) in 150 mM NaCl. pH adjusted with NaOH/HCl via automated titration.

The hydrodynamic diameter of the various polymers in solution was evaluated by dynamic light scattering (DLS) as a function of pH (Figure 2). The pH-dependent conformational change is well demonstrated through changes in the measured diameter. Decreasing the solution pH below the effective p*K*_a1_ of the respective polymers results in a notable increase in hydrodynamic diameter, indicative of the hydrophobically driven coil-to-globular transition resulting from the protonation of the diacid moieties.

### Solubilization of Model Lipid Membranes and Resultant Nanodisc Morphology

The effectiveness of polymer-mediated membrane solubilization can be influenced by various factors, where the membrane properties can affect the ability of the polymer to interact with and insert into the bilayer to create stable nanodiscs. DMPC LUVs or lipid-only systems are commonly used as simplified models to mimic the lipid bilayer structure of biological membranes. The BzAM series proved effective in solubilizing these synthetic membranes, where Figure S10 shows the average nanodisc diameter (nm) as a function of pH as evaluated by DLS.

Although there is no clear correlation between solution pH, polymer hydrophobicity, and resultant nanodisc size, some variation in size occurs under different pH conditions. The most hydrophilic SMA1:1 base copolymer generally shows much larger particles over the tested pH range. This can be ascribed to the highly hydrophilic nature of the copolymer.^48^ Additional analyses are required to determine whether the resultant nanodisc size is a consequence of overall polymer hydrophobicity. In the future, sample purification by gel filtration prior to DLS analysis will aid in acquiring more definitive size results.

The resultant nanodisc morphologies were confirmed using negative-stain transmission electron microscopy (TEM). Four BzAM samples (0.25–0.40 BzAM) were selected for gel filtration purification to obtain peak fractions of a monodispersed disc population of approximately 10 nm, as confirmed by DLS. Figure S11 shows the discoidal particles obtained.

### Divalent Cation Stability

Several biochemical or functional protein assays depend on divalent cations such as magnesium (Mg^2+^) or calcium (Ca^2+^).^18^ The diacid functionality of maleic acid can chelate with divalent cations, which can induce a conformational change in the nanodisc-stabilizing polymer and result in polymer precipitation and nanodisc instability.^49^ Therefore, it is necessary to evaluate the stability of the polymer-stabilized nanodiscs in the presence of these cations. Table 3 and Figure S12 show the maximum tolerable concentrations of Mg^2+^ and Ca^2+^ determined by optical density measurements.

**Table 3:** Summary of Polymer and Lipid-Only Polymer Nanodisc Properties under Various Biophysical Conditions Including pH, [Mg^2+^] and [Ca^2+^] (mM), and Average Lipid-Only Nanodisc Diameter (nm)

sample	soluble pH range^a^	[Mg^2+^] (mM)^b^	[Ca^2+^] (mM)^b^	average lipid-only nanodisc diameter (nm)^c^
SMA2:1	5.5–10	5	5	5.4 (±0.4)
SMA1:1	4–10	10	5	16.5 (±9.8)
0.05 BzAM	5–10	10	5	11.5 (±1.3)
0.10 BzAM	5–10	8	5	6.2 (±0.5)
0.15 BzAM	5.5–10	8	5	6.6 (±0.7)
0.20 BzAM	5.5–10	5	5	7.3 (±1.1)
0.25 BzAM	6–10	5	5	7.3 (±0.9)
0.30 BzAM	6–10	5	5	6.6 (±0.3)
0.35 BzAM	7–10	5	2	7.0 (±1.6)
0.40 BzAM	7–10	<5	2	7.2 (±0.9)

a Determined through DLS and effective p*K*_a_ measurements of polymer-only solutions, where marked increased hydrodynamic diameter (nm) is related to polymer aggregation and/or precipitation (0.5% (w/v) polymer).

b Optical density measurements at 630 nm evaluated the stability of DMPC lipid-only nanodiscs under varying divalent cation concentrations at pH 8.0. Increased optical density (>0.1 A.U.) is associated with nanodisc instability.

c Average DMPC lipid-only nanodisc diameter (nm) at pH 8.0, determined by DLS (data shown are mean ± SD (*n* = 3)), final polymer concentration of 2.5% (w/v) and 1.25 mg·mL^–1^ DMPC (16 h incubation at 25 °C).

Generally, increased sensitivity to divalent cations as a function of overall polymer hydrophobicity is observed. This is assigned to the relative decrease in the diacid moieties along the polymer backbone.^50^

### Solubilization and Purification of an MP from Biological Membranes

The solubilization performance of the terpolymers on a more complex and biologically representative membrane system was evaluated using the ATP-binding cassette (ABC) transporter, Sav1866, overexpressed in *E. coli*, as a MP prototype. Sav1866 is a bacterial homologue of the human multidrug resistance 1 gene (Mdr1) ABC transporter, which induces multidrug resistance in cancer cells.^51,52^ Sav1866 is a homodimeric ABC “half-transporter” with two identical subunits. Twelve elongated transmembrane (TM) helices form an extrusion cavity in the outward-facing conformation.

**Figure 3:**
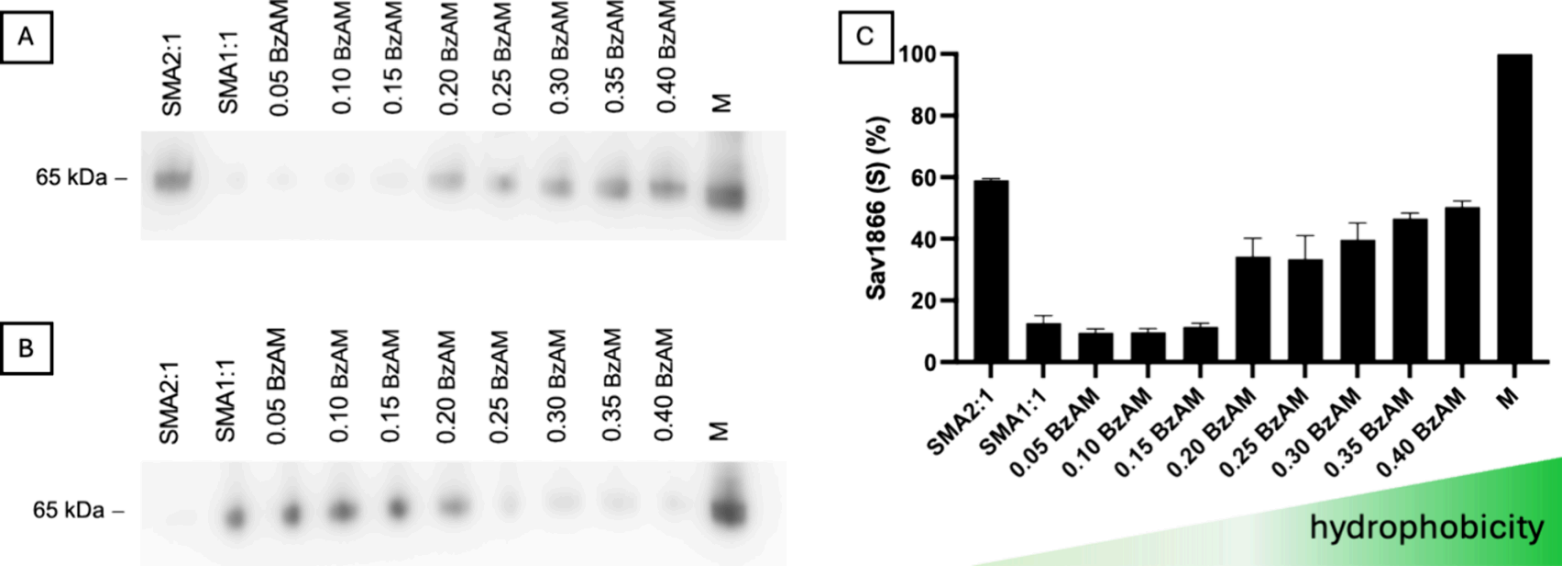
Representative Western blot of BzAM-Sav1866 using an anti-His antibody to detect His-tagged Sav1866. Crude membrane (M) was solubilized (2.5% (w/v) polymer, 2 h solubilization, ca. 25 °C) and underwent centrifugation (100,000 × *g*, 1 h, 4 °C) to generate (A) soluble (nanodiscs) fraction and (B) insoluble fraction. (C) Percentage Sav1866 detected in the soluble fraction (S) compared to the crude membrane fraction (M) was quantified through densitometric analysis. Data are mean ± SD (*n* = 2).

Figure 3 shows the antigen-specific Western blot (A, B) and densitometric analysis (C) for each BzAM terpolymer compared to the alternating SMA1:1 base copolymer and commercial SMA2:1. The density of the 65 kDa band is proportional to the abundance of the Sav1866 transporter solubilized from the membrane following incubation of the various polymer solutions with prepared *E. coli* membranes.

A very faint band corresponding to Sav1866 was observed in the SMA1:1 soluble fraction (Figure 3A), indicating poor target protein solubilization compared to the crude membrane fraction (M). This is not unexpected as SMA1:1 is known to exhibit poor performance in MP extraction.^34^ For the most hydrophilic terpolymers (0.05–0.15 BzAM), a low level of Sav1866 solubilization was observed, likely due to the low hydrophobicity of these terpolymer derivatives. However, for 0.20 BzAM, a noticeable increase in the soluble band density was observed. This improvement continued with increasing hydrophobicity of the subsequent BzAM derivatives. The soluble band density of the 0.30–0.40 BzAM derivatives aligns with that of SMA2:1, as anticipated, due to the comparable hydrophobic/hydrophilic balance of the polymers.

As solubilization efficiency increased, the density of the 65 kDa band in the soluble fraction (Figure 3A) increased with a corresponding decrease in the 65 kDa band in the insoluble fraction (Figure 3B). In the case of Sav1866, an evident correlation between polymer hydrophobicity and extracted protein yield was observed.

These results suggest that a fine balance exists between the hydrophilic/hydrophobic properties of solubilizing polymers and may be a crucial driving force for successful protein solubilization from biological membranes.

Furthermore, the BzAM series were assessed for their ability to extract, isolate and purify His-tagged Sav1866 using affinity chromatography. This small-scale experiment detected the eluted protein using Western blots, as standard protein stains lacked sufficient sensitivity. Despite lower relative yields compared to the crude solubilization (Figure 3 and Figure S13), Sav1866 was successfully detected in the eluted fractions (Figure S14). This indicates that the BzAM terpolymers are compatible with nickel affinity resin, facilitating the target protein’s binding and elution. Notably, following affinity purification, the outcome aligned with the previously observed trend, where increased polymer hydrophobicity corresponded to higher relative protein yields, likely reflecting the extent of Sav1866 solubilization by each polymer.

This study evaluated the effect of polymer amphiphilicity on protein extraction efficiency under typical solubilization conditions (i.e., 2.5% (w/v) polymer).^18^ To assess the impact of varying polymer concentration, the relative solubilization yield of two BzAM derivatives, 0.15 BzAM (less hydrophobic) and 0.35 BzAM (more hydrophobic) were assessed through immunoblotting, affinity purification, and subsequent densitometric analysis.

**Figure 4:**
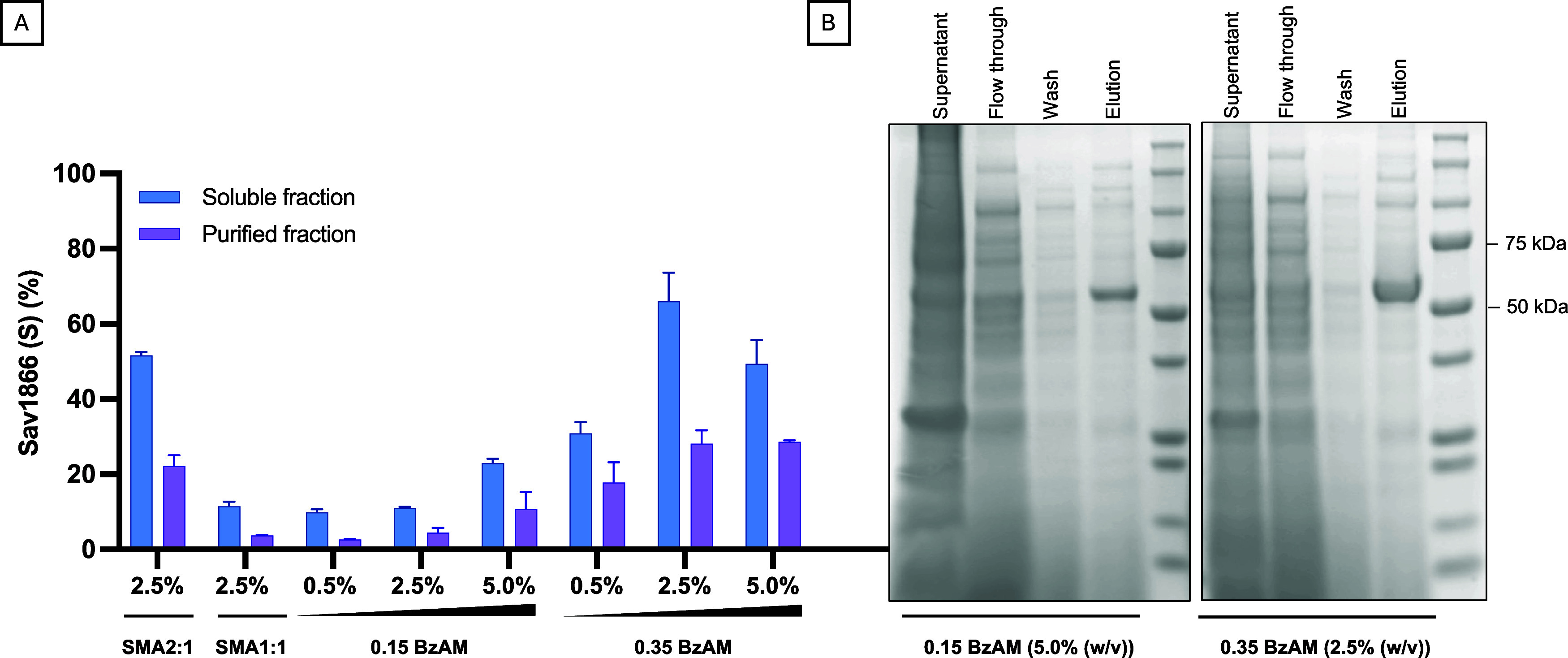
Solubilization and purification of Sav1866 using the BzAM terpolymers. (A) Effect of varying polymer concentrations (% (w/v)) on the percentage Sav1866 detected in the soluble fraction (S) (0.5, 2.5, or 5.0% (w/v) polymer, 2 h solubilization, ca. 25 °C) before and after affinity purification, quantified through densitometric analysis against the crude membrane fraction following immunoblotting. Data are mean ± SD (*n* = 2). (B) Coomassie-stained PAGE gel was used to assess the purification following polymer-mediated solubilization using 0.15 BzAM (5.0% (w/v) and 0.35 BzAM (2.5% (w/v)).

For the less hydrophobic derivative (0.15 BzAM), a higher polymer concentration (5.0% (w/v)) increased the target protein yield (Figure 4A). Conversely, the same concentration of the more hydrophobic derivative (0.35 BzAM) had an opposing effect, reducing the relative extraction yield compared to 2.5% (w/v) polymer (Figure 4A). The increased extraction yield at higher polymer concentrations for 0.15 BzAM aligns with previous reports on the more hydrophilic poly(diisobutylene-*alt*-maleic acid) (DIBMA) copolymer. Lower target protein yields were attributed to the reduced hydrophobicity of DIBMA compared to SMA2:1^53^ where increased DIBMA concentrations were required to enhance the yield for certain MPs.^27,54^ To confirm these observations, 0.15 BzAM and 0.35 BzAM at 5.0% (w/v)) and 2.5% (w/v)), respectively, were used in a larger-scale purification of Sav1866 (Figure 4B). Each polymer enabled the solubilization and affinity purification of the target protein, where the eluted fractions showed considerable enrichment of Sav1866.

Notably, in terms of final pure yield of Sav1866, the 0.35 BzAM polymer performed comparably to conventional SMA2:1 (at 2.5% (w/v)). Additional research is required to evaluate and optimize the binding, elution, and resulting purity of proteins solubilized by the different BzAM terpolymers. Nonetheless, these findings highlight the significance of optimizing polymer parameters for solubilization efficiency and downstream processing.

### Polymer Amphiphilicity

Membrane-solubilizing polymers require a considerable degree of amphiphilicity, i.e., adequate hydrophilicity to maintain aqueous solubility, and sufficient hydrophobicity to facilitate membrane insertion into the lipid core and form stable nanodiscs. The insertion of the polymer’s hydrophobic moieties weakens the hydrophobic interactions between the acyl lipid tails within the bilayer, allowing the polymer to disrupt the membrane and solubilize portions thereof into nanodiscs.^55^ Upon nanodisc formation, the amphiphilic polymer is positioned around the disc periphery, with the hydrophobic moieties orientated between the lipid acyl chains.^55^ As shown in this study, a careful balance is required between the hydrophobic and hydrophilic properties of the polymer. Excessive hydrophobicity can lead to the formation of aggregates and precipitation (e.g., 0.45 and 0.50 BzAM), whereas excessive hydrophilicity may result in poor solubilization efficiency, e.g., SMA1:1 and 0.05 BzAM.

An optimal hydrophobic/hydrophilic balance is imperative for efficient MP solubilization and stabilization. The delicate balance between these two opposing parameters is exemplified in the incremental variations observed in the BzAM terpolymer series. This is a noteworthy example of how a precisely tunable series of well-defined polymers can be useful in the development of protocols to investigate MP targets. We believe that the optimal hydrophobic/hydrophilic balance is influenced by both the membrane lipid composition and the target MP.

## Conclusions

This study establishes the utility of BzAM terpolymers for the solubilization and subsequent purification of MP encapsulated in a nanodisc. Unlike other polymers available, a systematic series of polymers has been generated and characterized, in which the hydrophilic/hydrophobic balance has been progressively changed. This will allow investigators to select the BzAM polymer with hydrophilic/hydrophobic characteristics best suited to encapsulating their specific MP of interest. The BzAM terpolymers described in this report bear similarities to existing commercial polymers with a critical distinction in their synthetic approach. Well-defined polymers characterized by predetermined molecular weights, narrow molecular weight distributions, and functional chain-end groups were created using controlled polymerization techniques, specifically RAFT-mediated polymerization.

The BzAM series was designed to emulate SMA2:1 while incorporating the benefits of a well-defined molecular architecture. The more hydrophobic BzAM derivatives exhibit a similar solution behavior, conditional constraints, and MP solubilization efficiencies to SMA2:1. This is the first report of the BzAM series’ unique tunable amphiphilicity enabling systematic investigation of the influence of polymer hydrophobic/hydrophilic balance on both polymer solution properties and membrane solubilization capabilities.

The solution behavior of the BzAM terpolymers was evaluated using potentiometric titrations, optical density measurements, and DLS to determine their stability under various pH conditions and divalent cation concentrations. Subsequently, their ability to solubilize synthetic lipid bilayers, and isolate and purify the 12TM Sav1866 from *E. coli* membranes was assessed. A direct relationship between polymer hydrophobicity and target protein solubilization was observed, with more hydrophobic BzAM derivatives solubilizing higher yields of Sav1866 (at 2.5% (w/v) polymer). As the terpolymers are derived from SMA1:1 without the *S*-butyl trithiocarbonate end group, their solution and solubilization properties are not influenced by chain length variations or the presence of an alkyl chain end. Variations in the polymer concentration influenced the relative MP extraction yield, where more hydrophilic derivatives may benefit from increased polymer concentrations. Overall, these findings emphasize the need for conditional optimization when evaluating polymer selection.

The progress made in broadening the capabilities and chemical diversity of polymers for membrane solubilization is promising for studying a wide range of MPs. Evidently, different polymers are better suited for different analyses, emphasizing the utility of well-defined polymer architectures with systematic modifications. These advancements can improve our understanding of their effects on synthetic and biological membrane solubilization, thereby facilitating improved polymer and protocol design. Furthermore, the benefits provided by RAFT-mediated polymerization, such as functional chain ends, enable future developments, including fluorescent or affinity tag-labeled polymers for advanced applications.

## Supporting Information Available

Synthetic details of BzAM synthesis; characterization of SMAnh, NMR parameters, and ^1^H and q-^13^C NMR spectra; TGA thermogram, SEC chromatograms, UV/vis, and ^1^H NMR spectra before and after end-group removal; characterization of the BzAM series via ^1^H NMR, ATR-FTIR, and q-^13^C NMR; ATR-FTIR spectra of the terpolymer series following hydrolysis; solution characterization via potentiometric titrations; characterization of lipid-only nanodisc size and morphology using DLS and negative-stain TEM; evaluation of lipid-only nanodisc stability under varying pH and divalent cation conditions; and Western blots for the densitometric analysis of Sav1866 extraction before and after Ni-NTA resin purification (PDF)

## Notes

*A patent application has been filed relating to the terpolymers described in this manuscript (WO 2023/115076).*

## Abbreviations

ABC transporter: ATP-binding cassette
AIBN: azobisisobutyronitrile
ATR: Attenuated total reflectance
BPT: *S*-butyl-*S*′-(1-phenyl ethyl) trithiocarbonate
BzAM: poly(styrene-*co*-maleic acid-*co*-(*N*-benzyl)maleimide
*Đ*: dispersity
DIBMA: poly(diisobutylene-*alt*-maleic acid)
DLS: dynamic light scattering
DMF: *N,N*-dimethylformamide
DMPC: 1,2-dimyristoyl-*sn*-glycero-3-phosphocholine
DMSO: dimethyl sulfoxide
*E. coli*: *Escherichia coli*
EG: end-group
EQP: equivalence point
ERC: equivalence recognition criterion
FTIR: Fourier transform infrared
LB: Luria broth
LDS: lithium dodecyl sulfate
LUV: large unilamellar vesicles
MAnh: maleic anhydride
Mdr1: multidrug resistance 1 gene
*M*_n_: number-average molecular weight
MP: membrane protein
MSM: maleic anhydride:styrene:maleic anhydride triad sequence
MSP: membrane-scaffold protein
*M*_w_: weight-average molecular weight
Ni-NTA: nickel(II)-nitrilotriacetic acid
NMR: nuclear magnetic resonance
PMMA: poly(methyl methacrylate)
PVDF: polyvinylidene fluoride
RAFT: reversible addition–fragmentation chain transfer
Sav1866: putative multidrug export ATP-binding/permease protein
PAGE: polyacrylamide gel electrophoresis
SEC: size exclusion chromatography
SMA: poly(styrene-*co*-maleic anhydride)
SMA1:1: poly(styrene-*alt*-maleic anhydride)
SMA2:1: poly(styrene-*co*-maleic anhydride) with 2:1 St:MAc
SMALP: styrene maleic acid lipid particle
SMAnh: poly(styrene-*alt*-maleic anhydride)
SSM: styrene:styrene:maleic anhydride triad sequence
SSS: styrene:styrene:styrene triad sequence
St: styrene
TEM: transmission electron microscopy
TGA: thermogravimetric analysis
TLC: thin-layer chromatography
TM: transmembrane
UV: ultraviolet
