# Supplementary material for: Tunable Terpolymer Series for the Systematic Investigation of Membrane Proteins
Source: Biomacromolecules. 2024 Dec 26;26(1):415–27. doi: 10.1021/acs.biomac.4c01219 (PMC11733950; doi:10.1021/acs.biomac.4c01219)
Supplement: Supplementary file 1 — bm4c01219_si_001.pdf [file bm4c01219_si_001.pdf]

Supplementary Information

Tuneable Terpolymer Series for the Systematic Investigation of Membrane Proteins

Authors: Gestél C. Kuyler [a,b], Elaine Barnard [a], Pooja Sridhar [c], Rebecca J. Murray [b,a], Naomi L. Pollock [c], Mark Wheatley [b,d], Timothy R. Dafforn [c], Bert Klumperman [*,a]

Affiliations:

[a] Department of Chemistry and Polymer Science, Stellenbosch University, Private Bag X1, Matieland, 7602, South Africa
[b] Centre for Health and Life Sciences, Coventry University, Coventry, CV1 2DS, UK
[c] School of Biosciences, University of Birmingham, Edgbaston, Birmingham, B15 2TT, UK
[d] Centre of Membrane Proteins and Receptors (COMPARE), University of Birmingham and University of Nottingham, Midlands, B15 2TT, UK

BzAM synthesis:

Table S1: Reagents and amounts (experimental and theoretical, respectively) used in the production of the BzAM series starting from SMAnh (12.0 g, 61.2 mmol MAnh) base copolymer and modifying with benzylamine (107.15 g/mol). $f_{MAnh(converted),theo}$ denotes the theoretical molar fraction of MAnh converted to N-benzyl maleimide derived from the experimental BzAM mole amounts ($n_{BzAM,exp}$), whereas $f_{MAnh(resultant),theo}$ denotes the theoretical molar fraction of residual MAnh after benzylamine modification.

Sample	***$Mass_{BzAM,exp}$***	***$n_{BzAM,exp}$***	***$f_{MAnh(converted),theo}$***	***$f_{MAnh(residual),theo}$***
	(g)	***(mmol)***		
0.05 BzAM	*0.333*	*3.11*	*0.05*	*0.95*
0.10 BzAM	*0.659*	*6.15*	*0.10*	*0.90*
0.15 BzAM	*0.993*	*9.27*	*0.15*	*0.85*
0.20 BzAM	*1.31*	*12.2*	*0.20*	*0.80*
0.25 BzAM	*1.64*	*15.3*	*0.25*	*0.75*
0.30 BzAM	*1.97*	*18.4*	*0.30*	*0.70*
0.35 BzAM	*2.30*	*21.5*	*0.35*	*0.65*
0.40 BzAM	*2.62*	*24.5*	*0.40*	*0.60*
0.45 BzAM	*2.95*	*27.5*	*0.45*	*0.55*
0.50 BzAM	*3.28*	*30.6*	*0.50*	*0.50*

NMR parameters:

Table S2: Experimental parameters pertaining to the respective NMR experiments.

	^{1}H-NMR	q^{13}C NMR
Number of scans	256	3584
Acquisition time (s)	3.99	1.37
Relaxation delay (s)	1.00	15.0
Pulse width (s)	1.00	15.0
Spectrometer frequency (MHz)	400.1	100.6
Temperature (K)	298	298

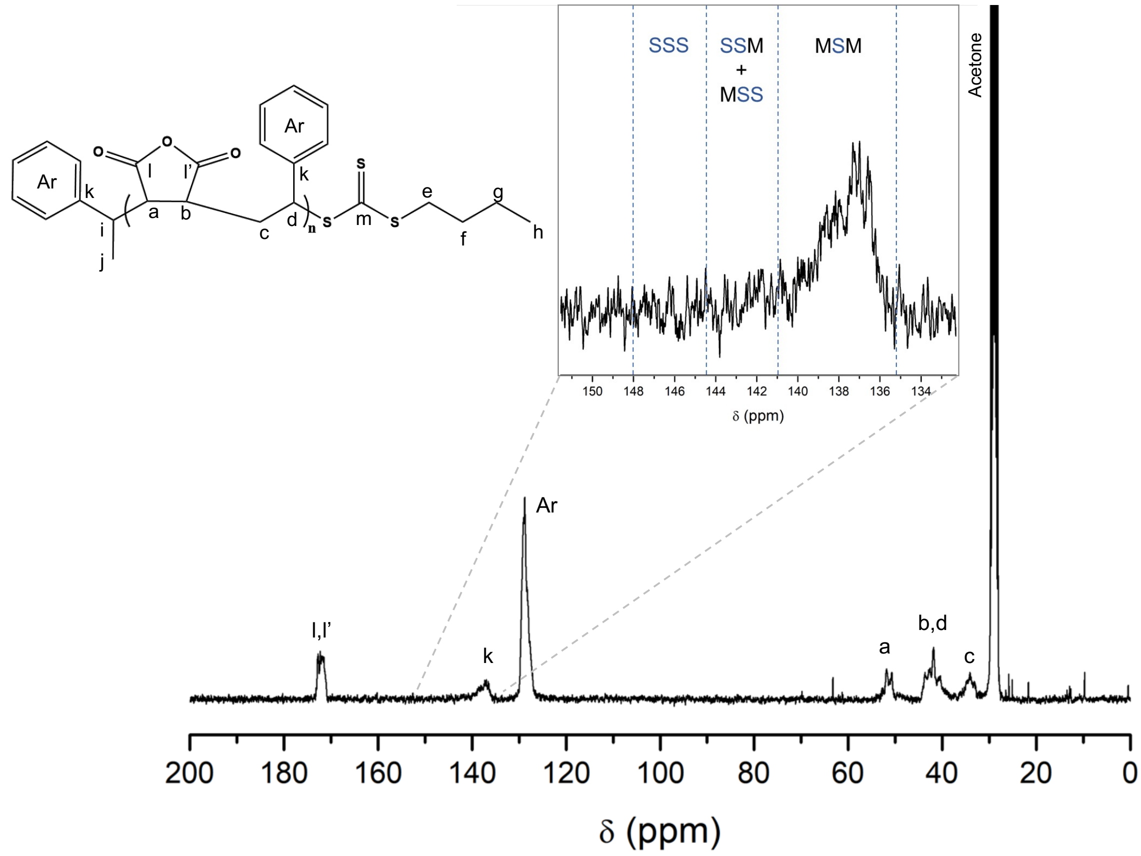

Figure S1: Quantitative ^{13}C-NMR (400 MHz, $(CD_3)_2CO$) spectrum of SMAnh where the figure insert highlights the chemical shift region of styrene's aromatic quaternary carbon (k) and the MSM microstructure derived from it.

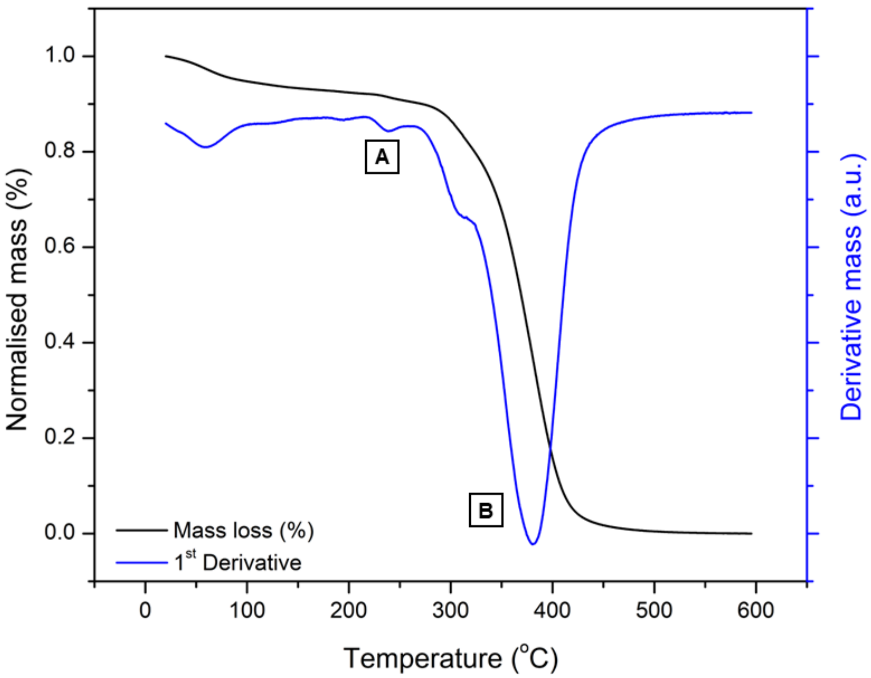

Figure S2: TGA thermogram of SMAnh shown as mass loss (%) (black) and its first derivative (blue) under nitrogen (N_2) gas flow in a temperature range from 25-600 °C at a constant heating rate of 10 °C/min. The peak of the calculated first derivative of the mass loss curve indicates the point of the greatest rate of change in the curve. Mass loss peak at (A) corresponds to the loss of the RAFT Z-group, while (B) corresponds to the onset of polymer degradation.

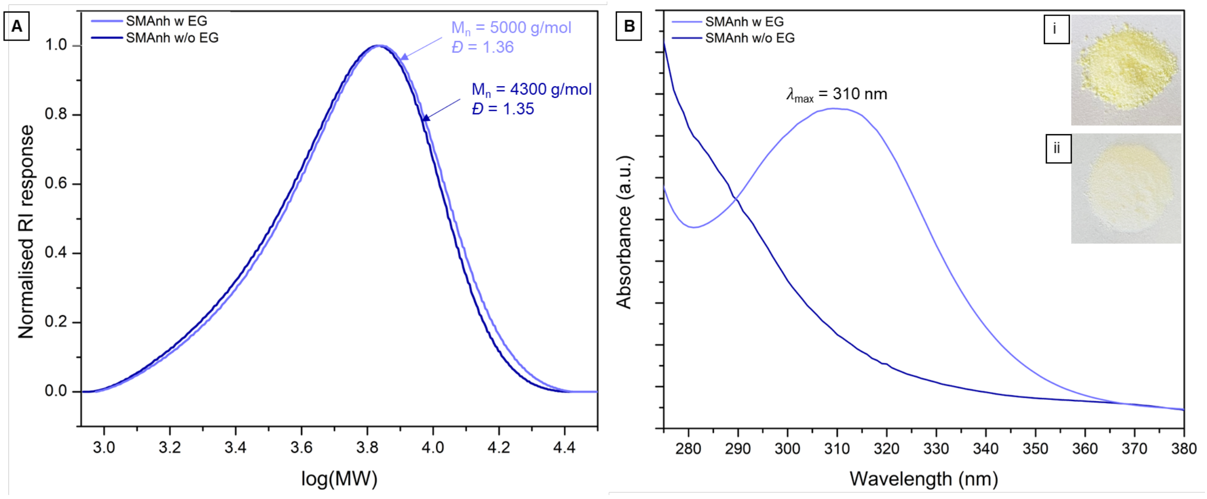

Figure S3: (A) Molecular weight distribution of SMAnh before and after end group removal; (B) UV absorbance spectra, and visual inspection (i) before and (ii) after thermolysis confirmed successful removal of the S-butyl trithiocarbonate end group (EG).

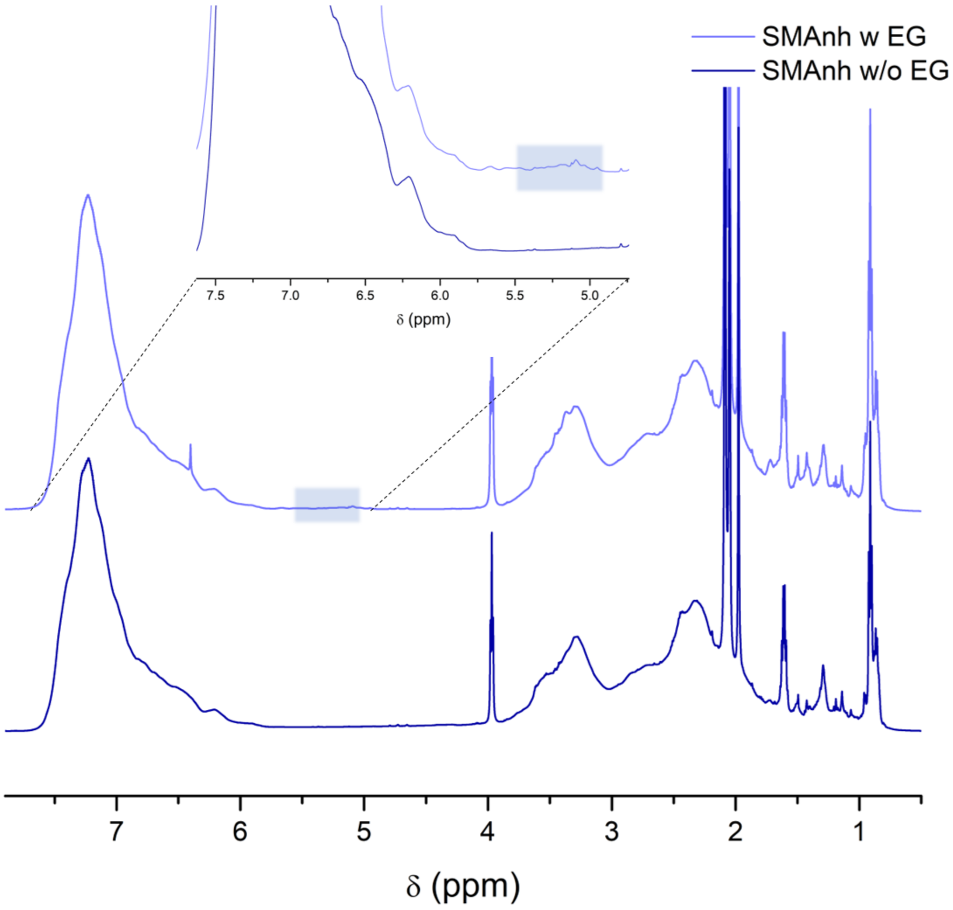

Figure S4: ^{1}H-NMR (400 MHz, $(CD_3)_2CO$) spectra of the SMAnh base polymer with (light blue) and without the end-group (blue) after thermolysis. The spectral insert indicates the broad peak (highlighted in blue) related to the terminal styrene monomer adjacent to the trithiocarbonate. The disappearance of this broad peak is indicative of the cleavage of the neighbouring trithiocarbonate moiety.

BzAM modification

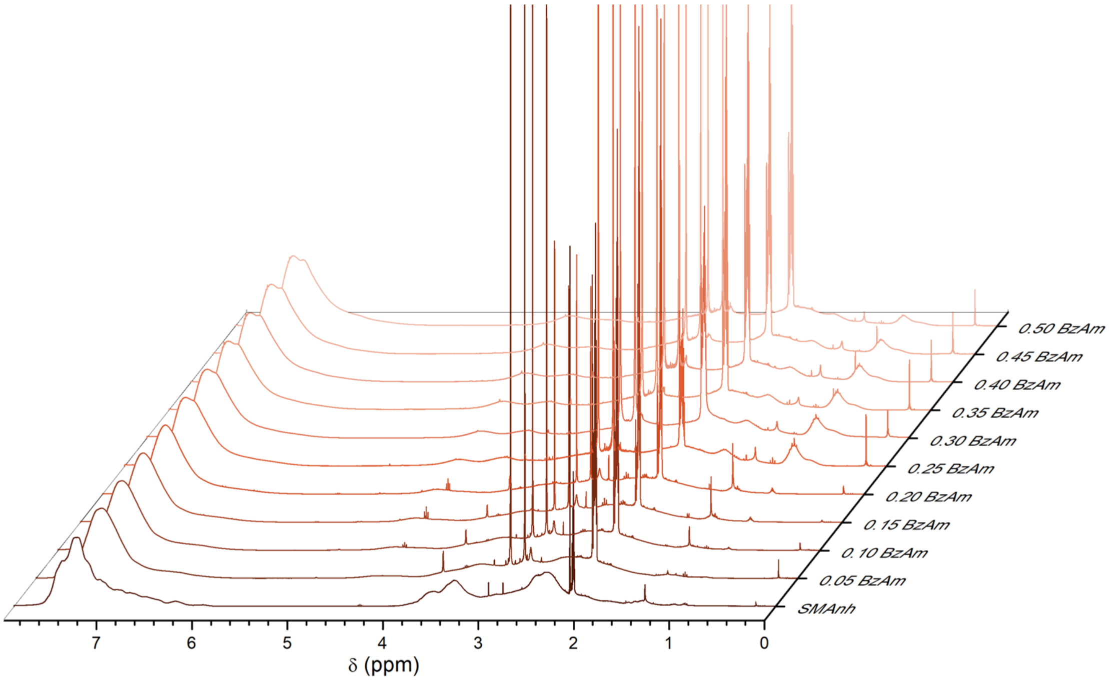

Figure S5: ^{1}H-NMR spectra of SMAnh (black)(400 MHz, $(CD_3)_2CO$) and the BzAM series (orange hues, where 0.05-0.20 BzAM, 400 MHz, $(CD_3)_2SO$) and (0.25-0.40 BzAM, 300 MHz, $(CD_3)_2CO$). Peak heights were normalised against the aromatic region at ~7.1 ppm. Notably, the appearance of the broad peak centred around 4.24 ppm corresponding to the methylene protons adjacent to the newly formed N-benzyl maleimides increases with increasing BzAM modification.

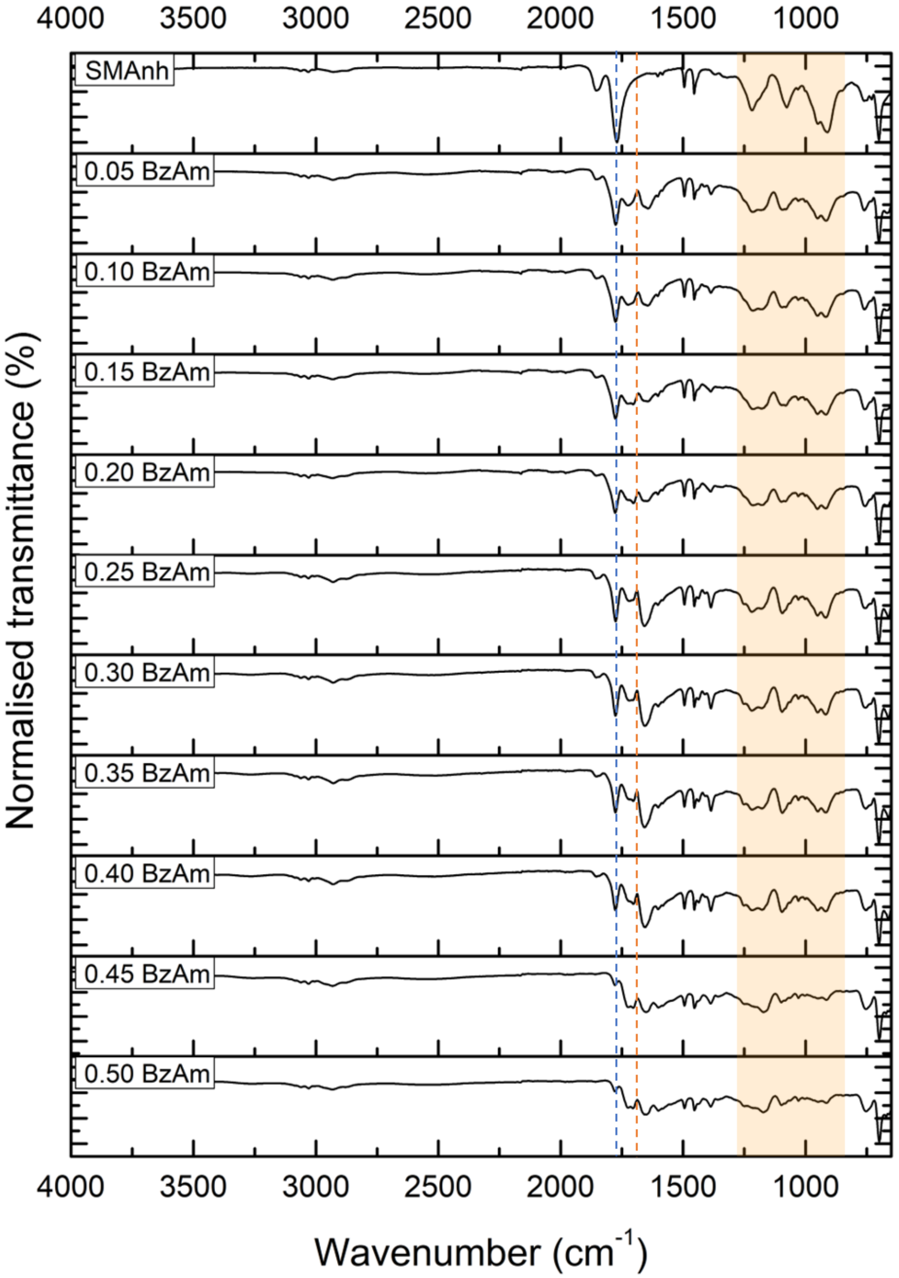

Figure S6: ATR-FTIR spectra of the SMAnh base copolymer (top panel) and the ring-closed BzAM terpolymer series. The stretching vibration range of the cyclic anhydride is highlighted in orange between 1285-860 cm^{-1}. Other peaks of interest include the cyclic anhydride asymmetric C=O stretch (blue dashed line at 1767 cm^{-1}).

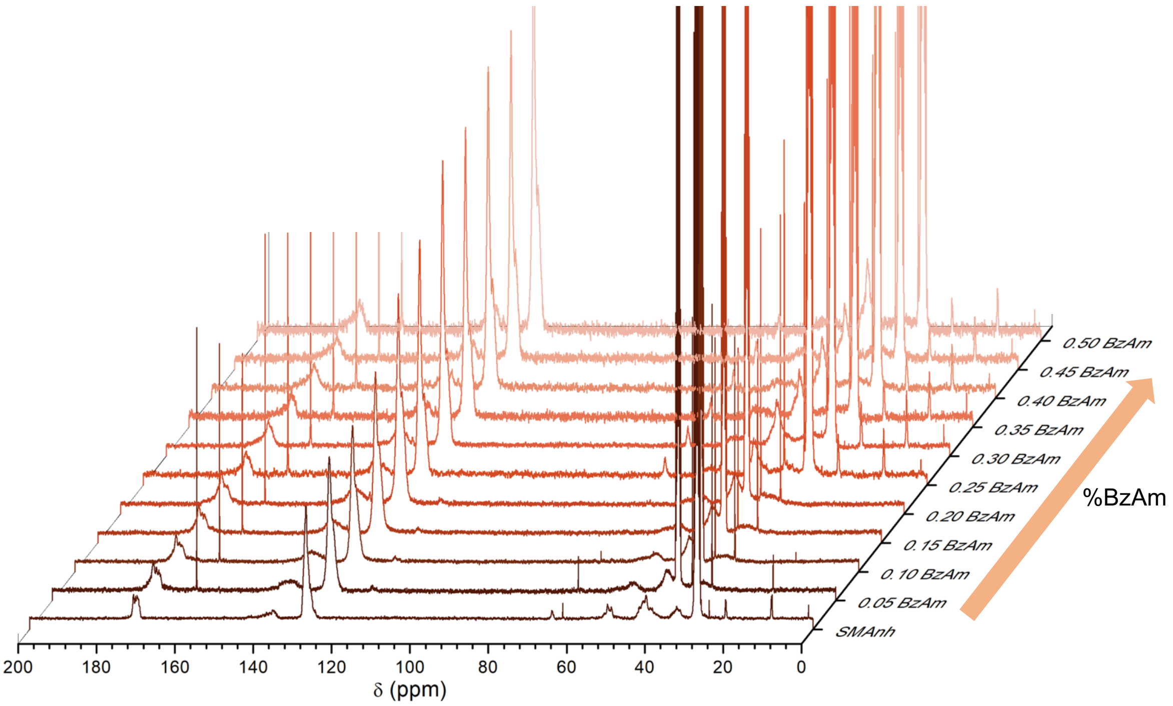

Figure S7: Quantitative ^{13}*C-NMR spectra of SMAnh (black, 400 MHz,* $(CD_3)_2CO$*) and the BzAM series (orange hues, where 0.05-0.20 BzAM (400 MHz,* $(CD_3)_2SO$*)) and 0.25-0.50 BzAM (300 MHz,* $(CD_3)_2CO$*). The orange arrow indicates the increase in the degree of modification (%BzAM). Peak heights were normalised against the carbonyl region at ~177 ppm.*
Relative peak integrations of the aromatic regions of SMAnh base copolymer (134-140 ppm) compared to the aromatic regions of the BzAM terpolymers (133-144 ppm) were used to calculate the degree of modification. Integrations are referenced against the aliphatic region of SMAnh (48-53 ppm) or BzAm derivative (46-57 ppm), respectively.

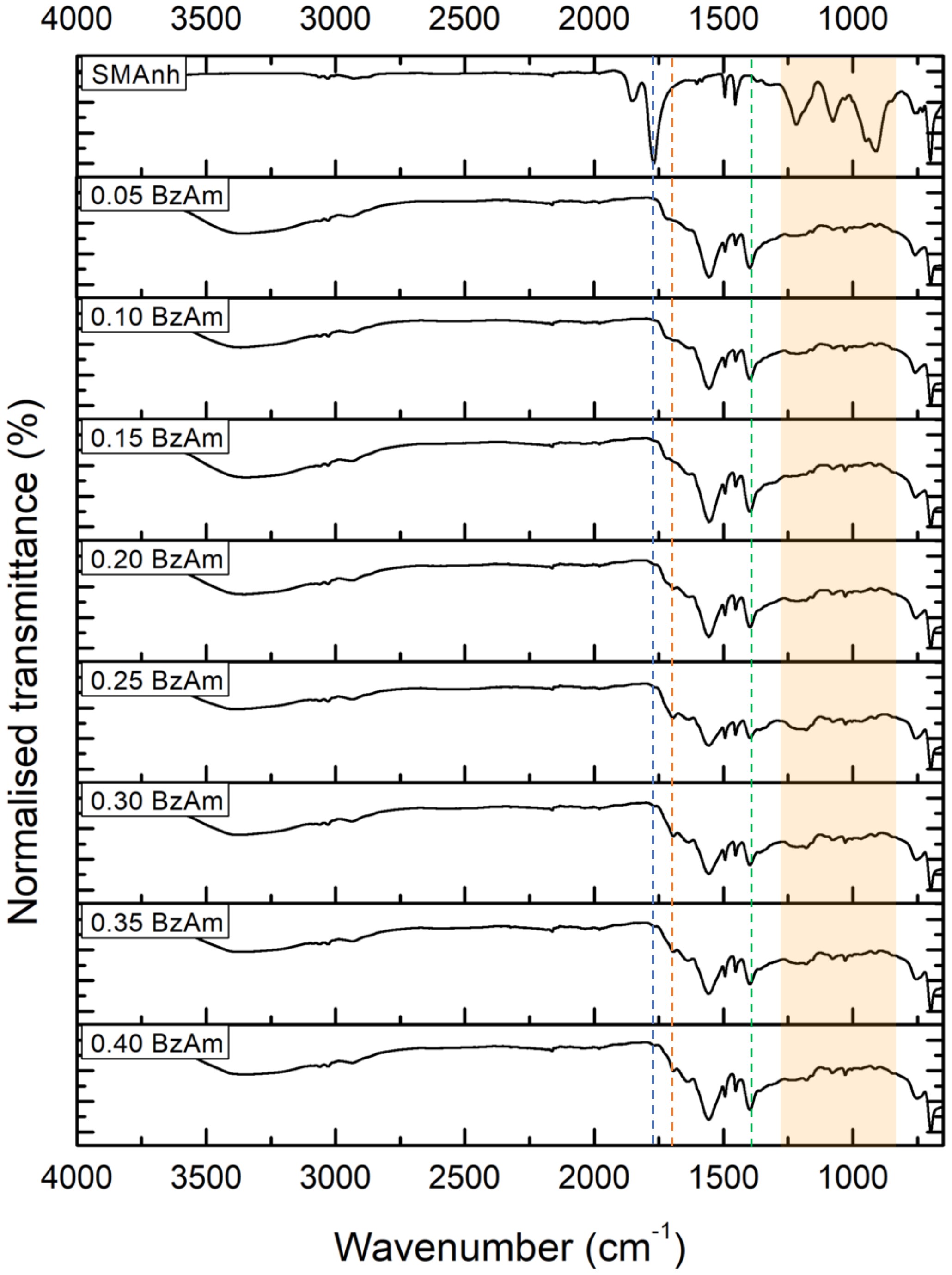

Figure S8: ATR-FTIR spectra of the ring-closed SMAnh base copolymer (top panel) and the hydrolysed, ring-opened BzAM terpolymer series. The lack of prominent peaks in the stretching vibration range of the cyclic anhydride is highlighted in orange between 1285-860 cm^{-1}. Other peaks of interest include the imide C-N stretch (green dashed line at 1387 cm^{-1}) and a broad O-H peak centred around 3400 cm^{-1} indicative of ring-opening hydrolysis.

Biophysical Characterisation

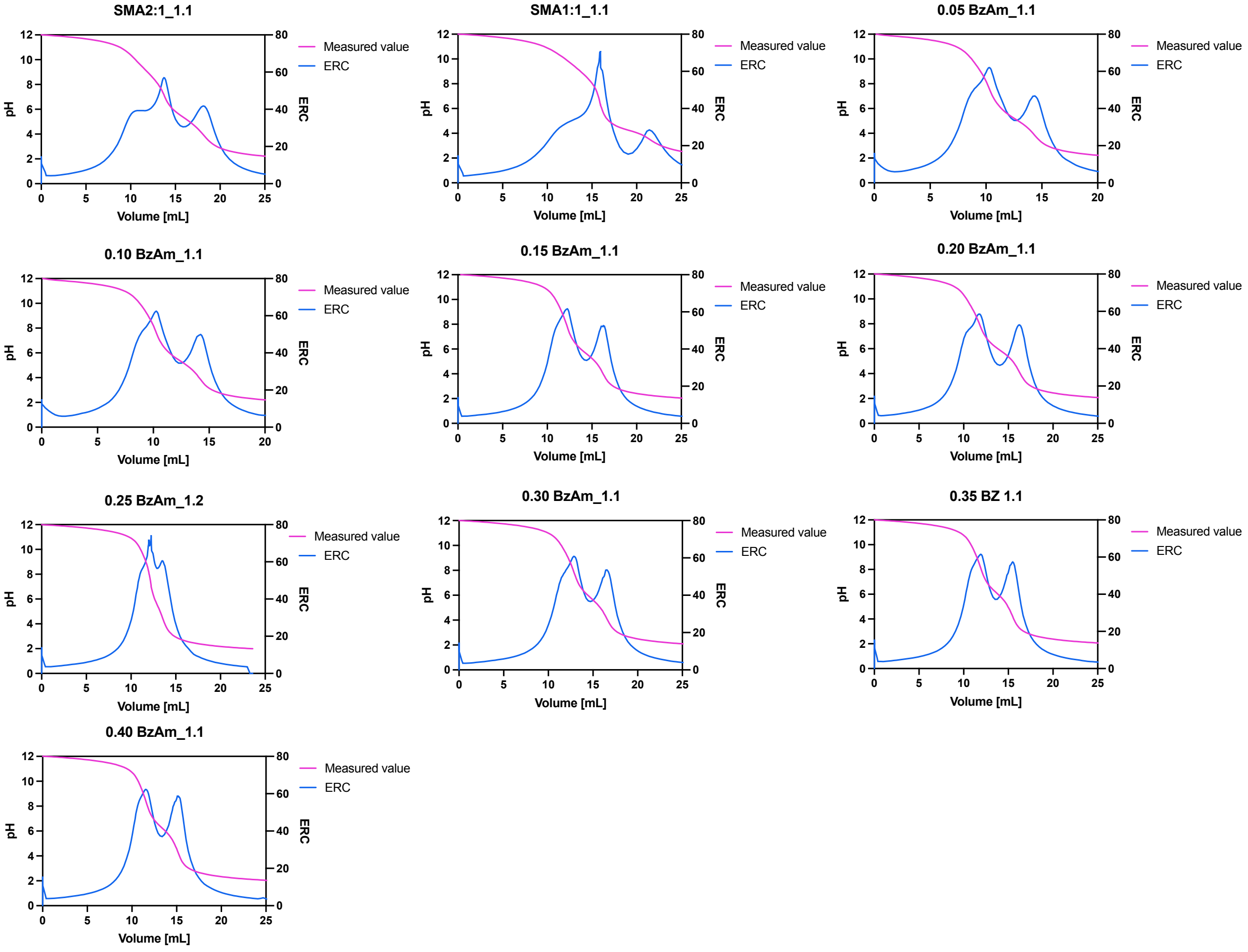

Figure S9: Representative potentiometric titration curves of SMA2:1, SMA1:1 and the BzAM series.

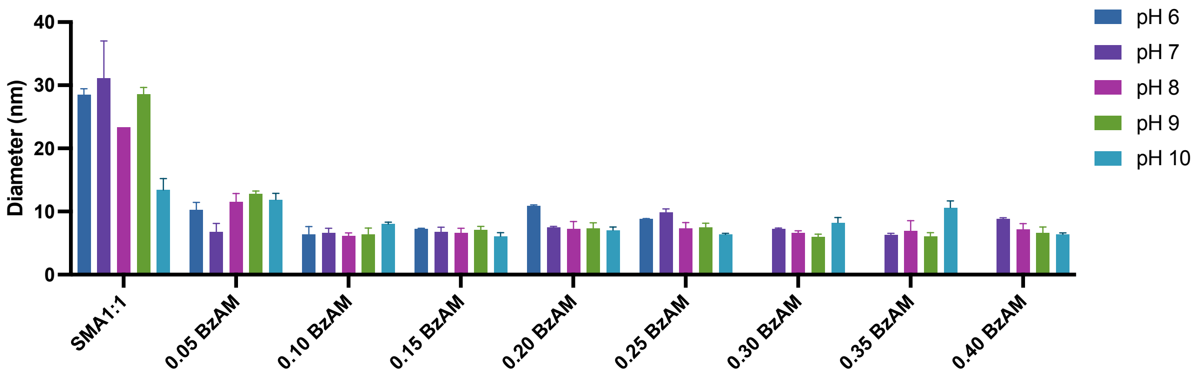

Figure S10: Average BzAM-stabilised DMPC nanodisc diameter (nm) as a function of pH determined by DLS. Lipid nanodiscs were formed using a final polymer concentration of 2.5 % (w/v) and 1.25 mg·mL^{-1}DMPC (16 h, ca. 25 °C).

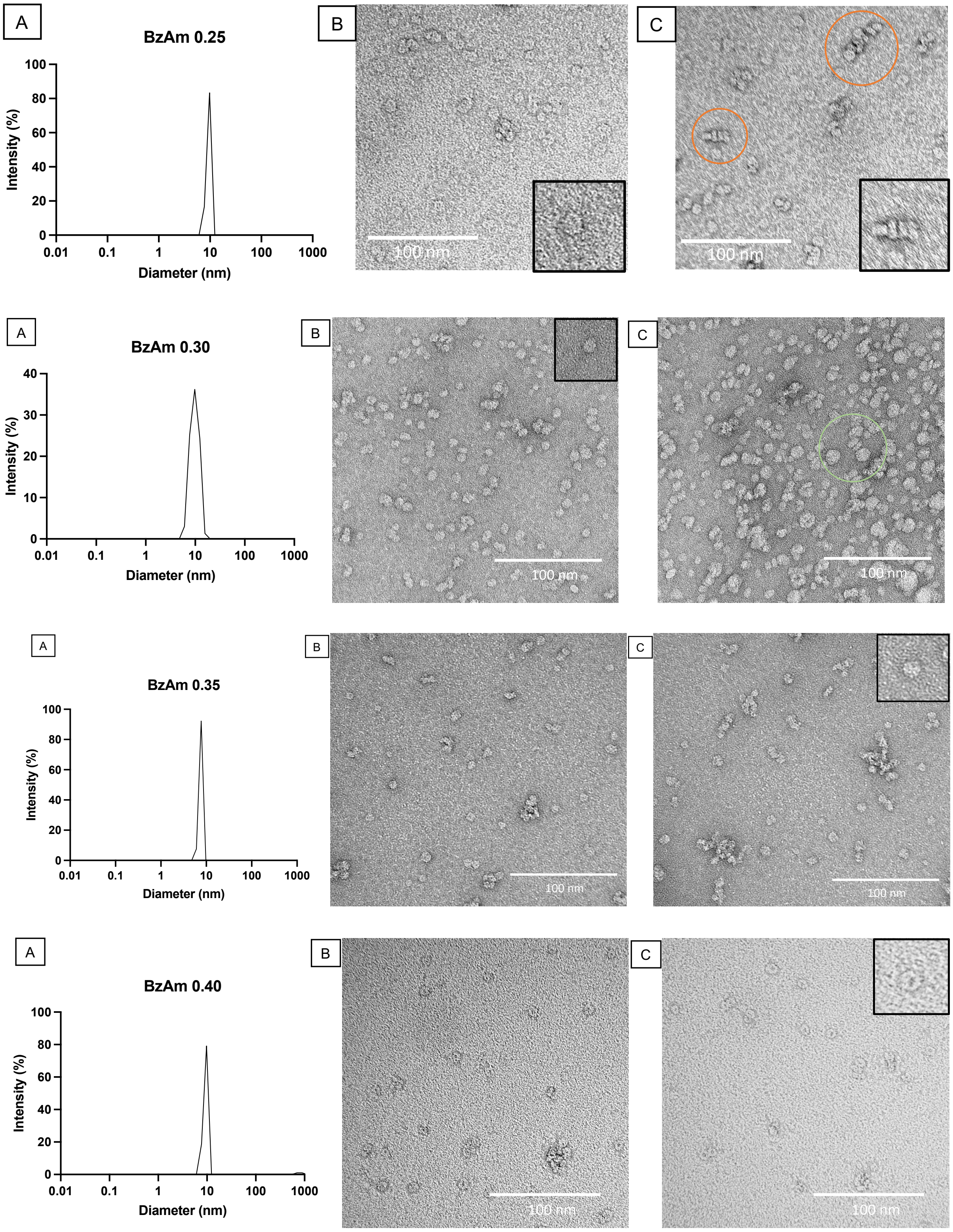

Figure S11: (A) DLS particle size determination as a proportion of intensity following gel filtration (Superdex 200 10/300 GL gel filtration column pre-equilibrated in 50 mM Tris, 150 mM NaCl, pH 7.4 at a flowrate of 0.5 mL/min). (B) and (C) Representative negative-stain TEM micrographs of BzAm-stabilised DMPC nanodiscs. Scale bars represent 100 nm at ×80,000 magnification. The insert of the figure shows top and encircled side views.

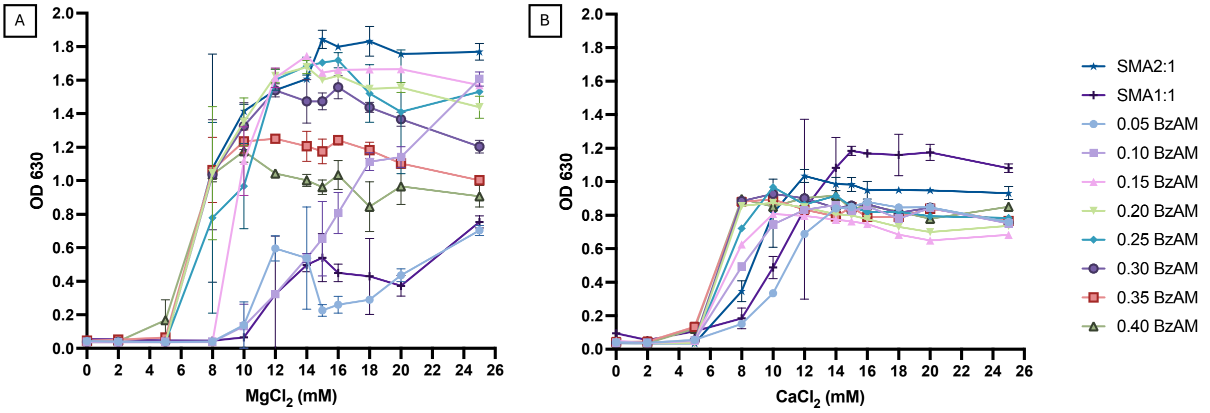

Figure S12: Optical density measurements at 630 nm to evaluate the stability of polymer-stabilised DMPC nanodiscs (50 mM Tris, pH 8.0, 0.5% (w/v) final polymer concentration, 1.25 mg·mL^{-1} DMPC) as a function of varying (A) $MgCl_2$ or (B) $CaCl_2$ concentrations. Increased optical density (>0.1 A.U.) is associated with polymer aggregation and/or precipitation.

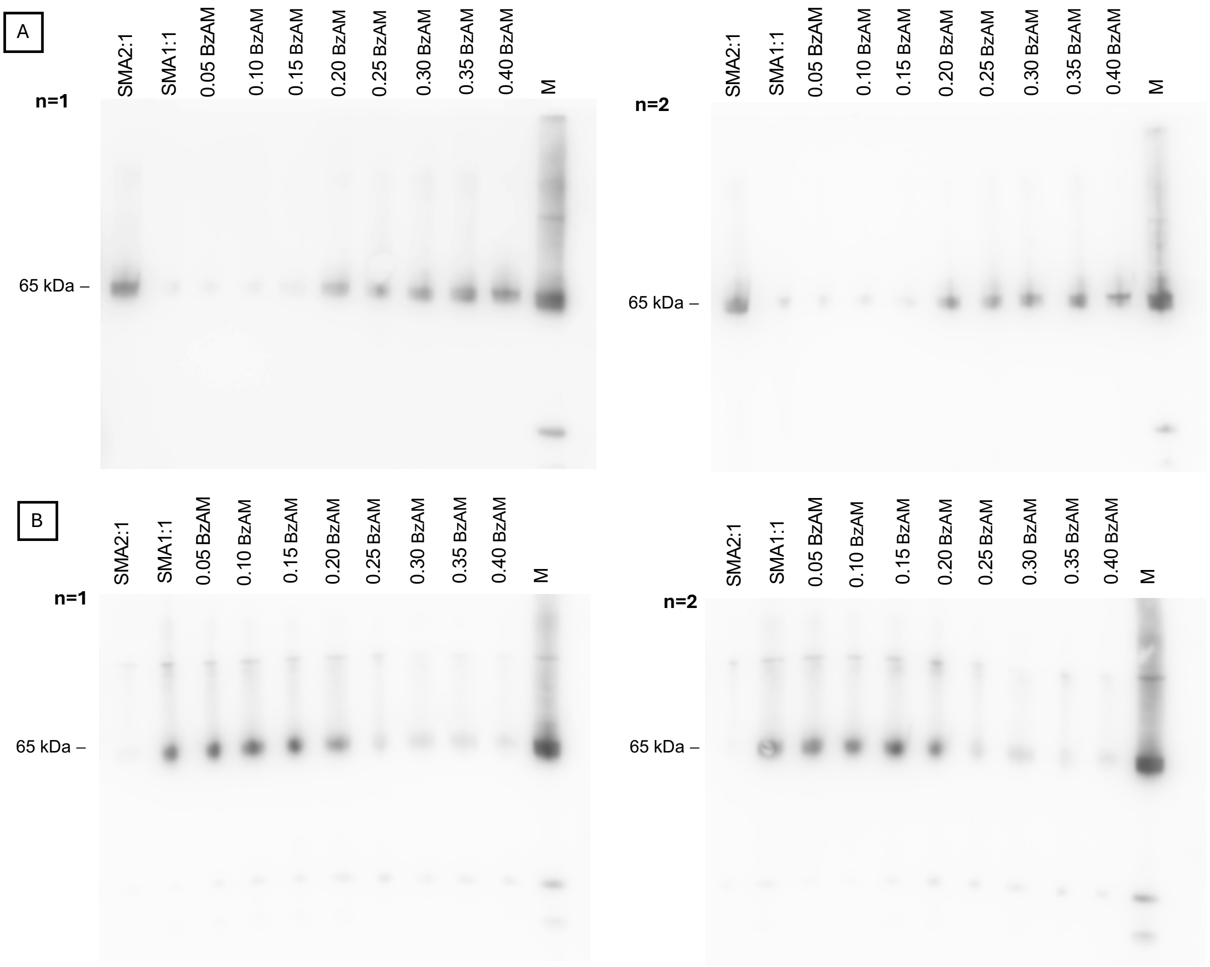

Figure S13: Western blots of BzAM-Sav1866 using anti-His antibody in the (A) soluble (nanodiscs) fraction and (B) insoluble fraction (2.5% (w/v) polymer, 2 h solubilisation, ca. 25 °C) used for densitometric quantification. Data are mean ± SD. (n = 2).

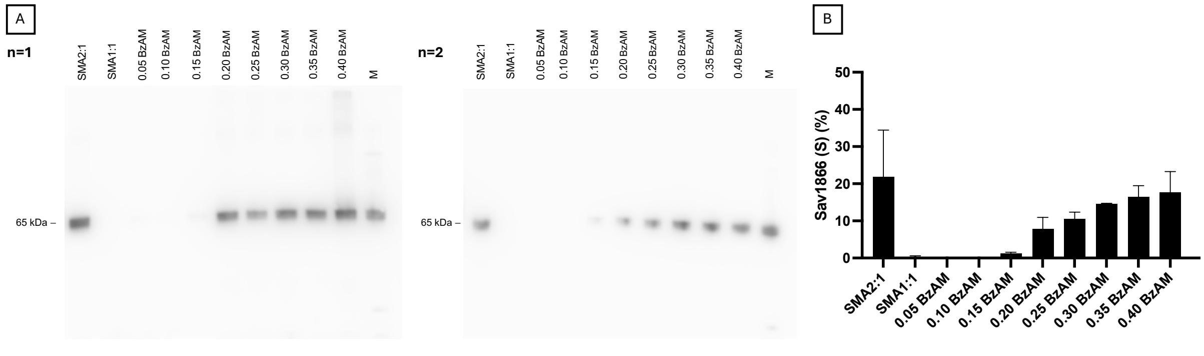

Figure S14: (A) Western blots of the BzAM-Sav1866 elution fractions using anti-His antibody following Ni-NTA resin purification (2.5% (w/v) polymer, 2 h solubilisation, ca. 25 °C) and (B) the percentage Sav1866 the original membrane preparation detected in the soluble fraction (S) compared to the crude membrane fraction (M); quantified through densitometric analysis. Data are mean ± SD. (n = 2).
